# Abstracts from the 23rd Italian congress of Cystic Fibrosis and the 13th National congress of Cystic Fibrosis Italian Society: Naples, Italy. 22-25 November 2017

**DOI:** 10.1186/s13052-017-0430-4

**Published:** 2018-01-16

**Authors:** Annamaria Bevivino, Alessandra Coiana, Annalisa Fogazzi, Fabiana Timelli, Sandra Signorini, Marco Lucarelli, Patrizia Morelli, Rita Padoan, Barbara Giordani, Annalisa Amato, Fabio Majo, Gianluca Ferrari, Serena Quattrucci, Laura Minicucci, Giovanna Floridia, Gianna Puppo Fornaro, Domenica Taruscio, Marco Salvatore, Manuela Seia, Silvia Pierandrei, Giovanna Blaconà, Valentina Salvati, Giovanni Sette, Giuseppe Cimino, Federica Sangiuolo, Adriana Eramo, Marco Lucarelli, Mirella Collura, Elisa Parisi, Annalisa Ferlisi, Gabriella Traverso, Marcella Bertolino, Lisa Termini, Maria A. Orlando, Caterina Di Girgenti, Valeria Pavone, Maria A. Calamia, Maria G. Silvestro, Caterina Lo Piparo, Francesca Ficili, Carla Colombo, Elizabeth Tullis, Jane C. Davies, Charlotte McKee, Cynthia DeSouza, David Waltz, Jessica Savage, Marc Fisher, Rebecca Shilling, Sam Moskowitz, Sarah Robertson, Simon Tian, Jennifer L. Taylor-Cousar, Steven M. Rowe, Elisa Beccia, Annalucia Carbone, Maria Favia, Stefano Castellani, Manuela Seia, Antonella Angiolillo, Carla Colombo, Valeria Casavola, Massimo Conese, Rita Padoan, Bruno M. Cesana, Diego Falchetti, Fiorella Battistini, Elisabetta Bignamini, Cesare Braggion, Mirella Collura, Natalia Cirilli, Maria C. Lucanto, Vincenzina Lucidi, Antonio Manca, Valeria Raia, Novella Rotolo, Donatello Salvatore, Sonia Volpi, Erica Nazzari, Riccardo Guarise, Palmiro Mileto, Francesca Garbarino, Gianfranco Alicandro, Alberto Battezzati, Carla Colombo, Barbara Giordani, Annalisa Amato, Fabio Majo, Gianluca Ferrari, Serena Quattrucci, Laura Minicucci, Rita Padoan, Giovanna Floridia, Gianna Puppo Fornaro, Domenica Taruscio, Marco Salvatore, Antonella M. Di Lullo, Marika Comegna, Felice Amato, Paola Iacotucci, Vincenzo Carnovale, Elena Cantone, Maurizio Iengo, Giuseppe Castaldo, Claudio Orlando, Alida Casale, Angela Sepe, Fabiola De Gregorio, Antonia De Matteo, Alice Castaldo, Chiara Cimbalo, Antonella Tosco, Valeria Raia, Daniela Savi, Serena Quattrucci, Michela Mordenti, Enea Bonci, Patrizia Troiani, Viviana D’Alù, Paolo Rossi, Monica Varchetta, Tamara Perelli, Serenella Bertasi, Paolo Palange, Giuseppe Cimino, Lucia Tardino, Giuseppe F. Parisi, Anna Portale, Chiara Franzonello, Maria Papale, Novella Rotolo, Salvatore Leonardi, Giuseppe F. Parisi, Lucia Tardino, Maria Papale, Chiara Franzonello, Francesca Pennisi, Novella Rotolo, Salvatore Leonardi, Sabina M. Bruno, Giulia Licciardello, Silvia Pierandrei, Giampiero Ferraguti, Serena Quattrucci, Marco Lucarelli, Giampiero Ferraguti, Manuela Sterrantino, Sabina M. Bruno, Silvia Pierandrei, Giancarlo Testino, Giuseppe Cimino, Serenella Bertasi, Marco Lucarelli, Donatello Salvatore, Rita Padoan, Roberto Buzzetti, Annalisa Amato, Barbara Giordani, Gianluca Ferrari, Fabio Majo, Cecilia Surace, Marco Lucarelli, Valentina M. Sofia, Fabio Majo, Giuseppe Cimino, Silvia Pierandrei, Nicola Ullmann, Vincenzina Lucidi, Antonio Novelli, Adriano Angioni, Marika Comegna, Antonella M. Di Lullo, Renato Liguori, Francesca Manzoni, Chiara Di Palma, Sabrina Maietta, Federica Zarrilli, Felice Amato, Valentina M. Sofia, Cecilia Surace, Vito Terlizzi, Federico Alghisi, Antonella Angiolillo, Cesare Braggion, Natalia Cirilli, Carla Colombo, Antonella M. Di Lullo, Rita Padoan, Serena Quattrucci, Valeria Raia, Giuseppe Tuccio, Federica Zarrilli, Antonio Novelli, Vincenzina Lucidi, Marco Lucarelli, Giuseppe Castaldo, Adriano Angioni, Valentina Tradati, Eliana di Stefano, Rita Padoan, Mirella Collura, Patrizia Dato, Maria G. Sciarrabone, Carmela Fondacaro, Lisa Termini, Annalisa Ferlisi, Maria A. Orlando, Gabriella Traverso, Marcella Bertolino, Francesca Ficili, Federico Cresta, Valentina Baglioni, Silvia Garuti, Isabella Buffoni, Francesca Landi, Rosaria Casciaro, Laura Minicucci, Daniela Girelli, Antonio Teri, Samantha Sottotetti, Arianna Biffi, Chiara Vignati, Monica D’accico, Anna Maraschini, Milena Arghittu, Carla Colombo, Giovanna Pizzamiglio, Elisa Cariani, Daniela Dolce, Novella Ravenni, Silvia Campana, Erica Camera, Carlo Castellani, Riccardo Guarise, Cesare Braggion, Giovanni Taccetti, Patrizia Morelli, Eleonora Calderone, Roberto Bandettini, Riccardo Guarise, Chiara Degli Innocenti, Chiara Castellani, Eleonora Masi, Vito Terlizzi, Maria Chiara Cavicchi, Beatrice Ferrari, Ramona Pezzotta, Piercarlo Poli, Serena Messali, Silvana Timpano, Rita Padoan, Erika Scaltriti, Stefano Pongolini, Simona Fiorentini, Silvia Bresci, Lorenzo Corsi, Riccardo Guarise, Beatrice Borchi, Annalisa Cavallo, Filippo Bartalesi, Massimo Pistolesi, Alessandro Bartoloni, Cesare Braggion, Mirella Collura, Francesca Ficili, Lisa Termini, Maria A. Orlando, Gabriella Traverso, Federica Arcoleo, Tiziana Pensabene, Marcella Bertolino, Maria A. Calamia, Annalisa Ferlisi, Annamaria Bevivino, Giovanni Bacci, Federica Armanini, Giovanni Taccetti, Vincenzina Lucidi, Daniela Dolce, Patrizia Morelli, Ersilia V. Fiscarelli, Nicola Segata, Alessio Mengoni, Barbara Giordani, Annalisa Amato, Fabio Majo, Gianluca Ferrari, Serena Quattrucci, Laura Minicucci, Rita Padoan, Giovanna Floridia, Gianna Puppo Fornaro, Domenica Taruscio, Marco Salvatore, Maria V. Di Toppa, Nicoleta Popa, Federico Alghisi, Francesco Felicetti, Sonia Graziano, Riccardo Ciprandi, Rita Pescini, Guendalina Graffigna, Serena Barello, Rosaria Casciaro, Federico Cresta, Laura Minicucci, Paola Catastini, Salvatore De Masi, C. Braggion, Maria A. Calamia, Annalisa Ferlisi, Maria G. Silvestro, Lucia Guarnuto, Francesca Ficili, Emanuela Di Liberti, Valentina Patti, Mirella Collura, Piercarlo Poli, Valentina Tradati, Rita Padoan, Massimo Luca Castellazzi, Valeria Daccò, Laura Claut, Carla Colombo, Matteo Giuliari, Luana Vicentini, Fausto Tilotta, Antonella Paciaroni, Sabino Della Sala, Cristina Guerzoni, Elisa Andreatta, Grazia Dinnella, Lisa Termini, Valeria Pavone, Elisa Parisi, Francesca Ficili, Maria A. Orlando, Annalisa Ferlisi, Gabriella Traverso, Orazia M. Granata, Tommaso S. Aronica, Mimì Crapisi, Donatella Fogazza, Marcella Bertolino, Mirella Collura, Mirella Collura, Luca Alessi, Flavia Mulè, Marcello Vitaliti, Mariarosaria Maresi, Maria A. Orlando, Annalisa Ferlisi, Gabriella Traverso, Lisa Termini, Marcella Bertolino, Francesca Ficili, Fabiola De Gregorio, Antonella Tosco, Alida Casale, Angela Sepe, Chiara Cimbalo, Andrea Catzola, Alice Castaldo, Laura Salvadori, Valeria Raia, Donatello Salvatore, Carmela Colangelo, Giovanni Marsicovetere, Michele D’Andria, Domenica Passarella, Carmela Genovese, Mirella Collura, Elisa Parisi, Annalisa Ferlisi, Mari A. Orlando, Gabriella Traverso, Lisa Termini, Marcella Bertolino, Caterina Di Girgenti, Maria A. Calamia, Maria G. Silvestro, Stefania Barrale, Maria R. Bonaccorso, Annalisa D’Arpa, Francesca Ficili

**Affiliations:** 1 ENEA Casaccia Research Center, Sustainable Territorial and Production Systems Department, Rome, Italy; 2 Dipartimento di Scienze Mediche e Sanità Pubblica, Università di Cagliari, Cagliari, Italy; 3 Cystic Fibrosis Support Centre, Department of Paediatric Children’s Hospital, ASST- Spedali Civili, Brescia, Italy; 4 Dept. of Cellular Biotechnologies and Hematology; Italian Pasteur Institute, Cenci Bolognetti Foundation, Sapienza University, Rome, Italy; 5 Laboratory Microbiology CF- Giannina Gaslini Institute-, Genoa, Italy; 6 Centro Regionale di Supporto per la Fibrosi Cistica, ASST Spedali Civili di Brescia, Brescia, Italy; 7 Lega Italiana Fibrosi Cistica ONLUS, Rome, Italy; 8 Unità Operativa Complessa Fibrosi Cistica Ospedale Pediatrico Bambino Gesù, Rome, Italy; 9 Centro Nazionale Malattie Rare, Istituto Superiore di Sanità, Rome, Italy; 10 Centro di Riferimento per la Fibrosi Cistica – Regione Lazio, Rome, Italy; 11 Centro di Riferimento per la Fibrosi Cistica – Regione Liguria, Genova, Italy; 12 Pre-BIO-Unità di bioetica, Istituto Superiore di Sanità, Rome, Italy; 13 Medical genetics laboratory, Fondazione IRCCS Ca’ Granda Ospedale Maggiore Policlinico, Milan, Italy; 14 Department of Cellular Biotechnologies and Hematology, Sapienza University of Rome, Rome, Italy; 15 Department of Pediatrics and Child Neuropsychiatry, Sapienza University of Rome, Rome, Italy; 16 Department of Oncology and Molecular Medicine, Italian Institute of Health, Rome, Italy; 17 Regional Reference Center for Cystic Fibrosis, Umberto I Hospital, Rome, Italy; 18 Department of Biomedicine and Prevention, Tor Vergata University of Rome, Rome, Italy; 19 Italian Pasteur Institute, Cenci Bolognetti Foundation, Sapienza University of Rome, Rome, Italy; 20 U.O. Pediatria II per la FIBROSI CISTICA (CRR) e le Malattie Respiratorie. ISMEP –, Palermo, Italy; 21 Dipartimento di Scienze per la promozione della salute materno infantile G. D’Alessandro. Università degli studi di Palermo, Palermo, Italy; 22 U.O.S.D. Genetica molecolare, ARNAS Civico, Palermo, Italy; 23 Fondazione IRCCS Ca’ Granda, Ospedale Maggiore Policlinico, University of Milan, Milan, Italy; 24 St Michael’s Hospital, Toronto, ON Canada; 25 Imperial College & Royal Brompton Hospital, London, UK; 26 Vertex Pharmaceuticals Incorporated, Boston, MA USA; 27 National Jewish Health, Denver, CO USA; 28 University of Alabama at Birmingham, Birmingham, AL USA; 29 Department of Medical and Surgical Sciences, University of Foggia, Foggia, Italy; 30 Department of Medicine and Health Sciences “V.Tiberio”, University of Molise, Campobasso, Italy; 31 Department of Bioscience, Biotechnology and Biopharmaceutics, University of Bari, Bari, Italy; 32 Medical Genetics Laboratory, Fondazione IRCCS Cà Granda Ospedale Maggiore Policlinico, Milan, Italy; 33 Cystic Fibrosis Center, Fondazione IRCCS Cà Grande Ospedale Maggiore Policlinico, Department of Pathophysiology and Transplantation, University of Milan, Milan, Italy; 34 Centro Regionale di Supporto per la Fibrosi Cistica, Clinica Pediatrica, Ospedale dei Bambini, ASST Spedali Civili Brescia, Brescia, Italy; 35 Dipartimento di Medicina molecolare e Traslazionale, Medicina e Chirurgia, Università di Brescia, Brescia, Italy; 36 Chirurgia Pediatrica, H Niguarda Ca’ Granda, Milan, Italy; 37 Centri Fibrosi Cistica di Brescia, Cesena, Italy; 38 Centri Fibrosi Cistica di Brescia, Torino, Italy; 39 Centri Fibrosi Cistica di Brescia, Firenze, Italy; 40 Centri Fibrosi Cistica di Brescia, Palermo, Italy; 41 Centri Fibrosi Cistica di Brescia, Ancona, Italy; 42 Centri Fibrosi Cistica di Brescia, Messina, Italy; 43 Centri Fibrosi Cistica di Brescia, Rome, Italy; 44 Italy Bambino Gesù, Bari, Italy; 45 Italy Bambino Gesù, Napoli, Italy; 46 Italy Bambino Gesù, Catania, Italy; 47 Italy Bambino Gesù, Potenza, Italy; 48 Italy Bambino Gesù, Verona, Italy; 49 Fondazione IRCCS Ca’ Granda Ospedale Maggiore Policlinico, Regional Cystic Fibrosis Center, Milan, Italy; 50 ICANS, Università degli Studi di Milano, Milan, Italy; 51 Dipartimento di Scienze Cliniche e di Comunità, Università degli Studi di Milano, Milan, Italy; 52 Lega Italiana Fibrosi Cistica ONLUS, Rome, Italy; 53 Unità Operativa Complessa Fibrosi Cistica Ospedale Pediatrico Bambino Gesù, Rome, Italy; 54 Centro Nazionale Malattie Rare, Istituto Superiore di Sanità, Rome, Italy; 55 Centro di Riferimento per la Fibrosi Cistica – Regione Lazio, Rome, Italy; 56 Centro di Riferimento per la Fibrosi Cistica – Regione Liguria, Genova, Italy; 57 Centro di Supporto per la Fibrosi Cistica – Regione Lombardia, Brescia, Italy; 58 Pre-BIO-Unità di bioetica, Istituto Superiore di Sanità, Rome, Italy; 59 Department of Molecular Medicine and Biotechnology, University of Naples Federico II, Naples, Italy; 60 CEINGE- Advanced Biotechnology, Naples, Italy; 61 Regional Center of Cystic Fibrosis, Adult Section, Department of Medical Traslational Science, University of Naples Federico II, Naples, Italy; 62 Department of Neuroscience, ENT Section, University of Naples Federico II, Naples, Italy; 63 Department of “Endoscopia Respiratoria Pediatrica e Vie Aeree Difficili”, AORN Santobono-Pausilipon, Naples, Italy; 64 Cystic Fibrosis Center, Department of Translational Medical Sciences University of Naples “Federico II”, Naples, Italy; 65 Department of Public Health and Infectious Diseases, Adult Cystic Fibrosis Center, “Sapienza” University of Rome, Rome, Italy; 66 Cystic Fibrosis Unit, Bambino Gesù Children’s Hospital, Rome, Italy; 67 Department of Pediatrics, Cystic Fibrosis Center, “Sapienza” University of Rome, Rome, Italy; 68 Department of Sperimental Medicine, “Sapienza” University of Rome, Rome, Italy; 69 U.O.C. Broncopneumologia Pediatrica e Fibrosi Cistica, University of Catania, Catania, Italy; 70 Department of Clinical and Experimental Medicine, University of Catania –, Catania, Italy; 71 Department of Cellular Biotechnologies and Hematology, Sapienza University of Rome, Rome, Italy; 72 Department of Pediatrics and Child Neuropsychiatry, Sapienza University of Rome, Rome, Italy; 73 Regional (Lazio) Reference Center for Cystic Fibrosis, Umberto I Hospital, Rome, Italy; 74 Italian Pasteur Institute, Cenci Bolognetti Foundation, Sapienza University of Rome, Rome, Italy; 75 Department of Cellular Biotechnologies and Hematology, Sapienza University of Rome, Rome, Italy; 76 Department of Pediatrics and Child Neuropsychiatry, Sapienza University of Rome, Rome, Italy; 77 Regional (Lazio) Reference Center for Cystic Fibrosis, Umberto I Hospital, Rome, Italy; 78 Italian Pasteur Institute, Cenci Bolognetti Foundation, Sapienza University of Rome, Rome, Italy; 79 Centro Regionale Fibrosi Cistica, AOR Ospedale San Carlo, Potenza, Italy; 80 Centro regionale di supporto per la fibrosi cistica, ASST Spedali Civili, Brescia, Italy; 81 Pediatra, epidemiologo, Bergamo, Italy; 82 Italian Cystic Fibrosis Registry, Rome, Italy; 83 Lega Italiana Fibrosi Cistica ONLUS, Rome, Italy; 84 Centro Nazionale Malattie Rare, Istituto Superiore di Sanità, Rome, Italy; 85 Centro Fibrosi Cistica, Ospedale Pediatrico Bambino Gesù, Rome, Italy; 86 UOC Laboratorio Genetica Medica, Ospedale Pediatrico “Bambino Gesù”, Rome, Italy; 87 Dip. di Biotecnologie Cellulari ed Ematologia, Sapienza Università di Roma, Rome, Italy; 88 Istituto Pasteur Fondazione Cenci Bolognetti, Sapienza Università di Roma, Rome, Italy; 89 UOC Fibrosi Cistica, Ospedale Pediatrico “Bambino Gesù”, Rome, Italy; 90 Centro di Riferimento Regionale Fibrosi Cistica, Azienda Policlinico Umberto I, Rome, Italy; 91 Centro di Riferimento Regionale Fibrosi Cistica, Azienda Policlinico Umberto I, Rome, Italy; 92 UO Broncopneumologia, Ospedale Pediatrico “Bambino Gesù”, Rome, Italy; 93 Department of Molecular Medicine and Biotechnology, University of Naples Federico II, Naples, Italy; 94 CEINGE- Advanced Biotechnology, Naples, Italy; 95 Department of Biosciences and Territory, University of Molise, Isernia, Italy; 96 Department of Neuroscience, ENT Section, University of Naples Federico II, Naples, Italy; 97 UOC Laboratorio Genetica Medica, Ospedale Pediatrico “Bambino Gesù”, Rome, Italy; 98 Dipartimento di Pediatria, Centro Regionale Toscano per la Fibrosi Cistica, Azienda Ospedaliero-Universitaria Meyer, Florence, Italy; 99 UOC Fibrosi Cistica, Ospedale Pediatrico “Bambino Gesù”, Rome, Italy; 100 Centre for Research and Training in Medicine for Aging, Department of Medicine and Health Sciences “Vincenzo Tiberio”, University of Molise, Campobasso, Italy; 101 Cystic Fibrosis Center, Anna Meyer Children’s Hospital, Florence, Italy; 102 Dipartimento Materno-Infantile, Ospedali Riuniti Ancona, Centro Regionale Fibrosi Cistica, Ancona, Italy; 103 Centro Regionale Fibrosi Cistica, Fondazione IRCCS Ca’ Granda, Ospedale Maggiore Policlinico, Università degli Studi di Milano, Milan, Italy; 104 CEINGE-Biotecnologie Avanzate, Naples, Italy; 105 Dipartimento di Medicina Molecolare e Biotecnologie Mediche, Università di Napoli Federico II, Naples, Italy; 106 Dipartimento di Neuroscienze, Sezione di ORL, Università di Napoli Federico II, Naples, Italy; 107 Cystic Fibrosis Support Center, Paediatric Department, Children’s Hospital, AO Spedali Civili, Brescia, Italy; 108 Centro Fibrosi Cistica, Sapienza Università e Policlinico Umberto I, Rome, Italy; 109 Centro Regionale Fibrosi Cistica, Sezione Pediatrica, Dipartimento di Scienze Mediche Traslazionali, Università di Napoli Federico II, Naples, Italy; 110 Centro di Riferimento per la Fibrosi Cistica - Regione Calabria, Soverato, Italy; 111 Dipartimento di Bioscienze e Territorio, Università del Molise, Isernia, Italy; 112 Dip. di Biotecnologie Cellulari ed Ematologia, Sapienza Università di Roma, Rome, Italy; 113 Istituto Pasteur Fondazione Cenci Bolognetti, Sapienza Università di Roma, Rome, Italy; 114 Cystic Fibrosis Support Centre, Department of Paediatric Children’s Hospital, ASST- Spedali Civili, Brescia, Italy; 115 U.O. Pediatria II per la FIBROSI CISTICA (CRR) e le Malattie Respiratorie. ISMEP, Palermo, Italy; 116 Dipartimento di Scienze per la promozione della salute materno infantile G. D’Alessandro. Università degli studi di Palermo, Palermo, Italy; 117 Cystic Fibrosis Center, Pneumology Unit, IRCCS G. Gaslini Institute, Genova, Italy; 118 Pneumology Unit, Policlinico San Martino - IST, Genova, Italy; 119 UOS Microbiology and Cystic Fibrosis Microbiology, Fondazione IRCCS Ca’ Granda Ospedale Maggiore Policlinico, Milano, Italy; 120 UOC Fibrosi Cistica Pediatrica, Cystic Fibrosis Centre, Fondazione IRCCS Ca’ Granda Ospedale Maggiore Policlinico, Milano, Italy; 121 Respiratory Disease Department, Cystic Fibrosis Center Adult Section, Fondazione IRCCS Ca’ Granda Ospedale Maggiore Policlinico Milano, Milano, Italy; 122 Cystic Fibrosis Centre, Department of Pediatric Medicine, Meyer Children Hospital, Florence, Italy; 123 Rehabilitation Unit, Meyer Children Hospital, Florence, Italy; 124 Microbiology Laboratory of CF - Giannina Gaslini Institute, Genoa, Italy; 125 Rehabilitation Unit, Meyer Children Hospital, Florence, Italy; 126 Cystic Fibrosis Centre, Department of Pediatric Medicine, Meyer Children Hospital, Florence, Italy; 127 Section of Microbiology, Department of Molecular and Translational Medicine University of Brescia/ASST - Spedali Civili, Brescia, Italy; 128 Cystic Fibrosis Regional Support Centre, Children Hospital, ASST - Spedali Civili, Brescia, Italy; 129 Istituto Zooprofilattico Sperimentale della Lombardia e dell’Emilia Romagna (IZSLER) “Bruno Ubertini” of Parma, Parma, Italy; 130 Tropical and Infectious Disease Department, Careggi, Florence, Italy; 131 Pneumology, Thoracic and Pulmonary Physiopathology Department, Careggi, Florence, Italy; 132 Rehabilitation Unit, Meyer Children Hospital, Florence, Italy; 133 Cystic Fibrosis Centre, Meyer Children Hospital, Florence, Italy; 134 U.O. di Pediatria per la Fibrosi Cistica e le malattie respiratorie. ISMEP (Palermo), Palermo, Italy; 135 Dipartimento di Scienze per la promozione della salute materno infantile G. D’Alessandro. Università degli studi di Palermo, Palermo, Italy; 136 U.O. C. Microbiologia. ARNAS Civico Palermo, Palermo, Italy; 137 ENEA Casaccia Research Center, Sustainable Territorial and Production Systems Department, Rome, Italy; 138 University of Florence, Department of Biology, Florence, Italy; 139 University of Trento, Centre for Integrative Biology, Trento, Italy; 140 Cystic Fibrosis Center, Anna Meyer Children’s University Hospital, Department of Pediatrics Medicine, Florence, Italy; 141 Children’s Hospital and Research Institute Bambino Gesù, CF Microbiology and CF Center, Rome, Italy; 142 Cystic Fibrosis Center, G. Gaslini Institute, Department of Pediatrics, Genoa, Italy; 143 Lega Italiana Fibrosi Cistica ONLUS, Roma, Italy; 144 Unità Operativa Complessa Fibrosi Cistica Ospedale Pediatrico Bambino Gesù, Roma, Italy; 145 Centro Nazionale Malattie Rare, Istituto Superiore di Sanità, Roma, Italy; 146 Centro di Riferimento per la Fibrosi Cistica – Regione Lazio, Roma, Italy; 147 Centro di Riferimento per la Fibrosi Cistica – Regione Liguria, Genova, Italy; 148 Centro di Supporto per la Fibrosi Cistica – Regione Lombardia, Brescia, Italy; 149 Pre-BIO-Unità di bioetica, Istituto Superiore di Sanità, Roma, Italy; 150 Cystic Fibrosis Unit, Bambino Gesù Pediatric Hospital, Rome, Italy; 151 Clinical Psychology Unit, Bambino Gesù Pediatric Hospital, Rome, Italy; 152 Università Cattolica del Sacro Cuore, Milano, Italy; 153 Istituto Giannina Gaslini, Genova, Italy; 154 CF Centre Meyer Children’s Hospital, Florence, Italy; 155 Clinical Trial Office Meyer Children’s Hospital, Florence, Italy; 156 U.O. II Pediatria per la Fibrosi Cistica e le malattie respiratorie. ISMEP, Palermo, Italy; 157 Università degli studi di Palermo, Palermo, Italy; 158 Cystic Fibrosis Support Center, Department of Pediatrics, Children’s Hospital, ASST Spedali Civili, Brescia, Italy; 159 Cystic Fibrosis Centre, Fondazione IRCCS Ca’ Granda, Ospedale Maggiore Policlinico, University of Milan, Milan, Italy; 160 U.O. di Pediatria, Centro di Supporto provinciale per la cura della Fibrosi Cistica, Ospedale di Rovereto, Trento, Italy; 161 U.O. di Radiologia, Ospedale di Rovereto, Trento, Italy; 162 Scuola di specializzazione in Pediatria. Università degli studi di Palermo, Palermo, Italy; 163 U.O. di Pediatria per la Fibrosi Cistica e le malattie respiratorie. ISMEP, Palermo, Italy; 164 U.O. Patologia clinica pediatrica. Screening neonatale metabolico allargato. ISMEP, Palermo, Italy; 165 U.O. di Pediatria per la Fibrosi Cistica e le malattie respiratorie. ISMEP, Palermo, Italy; 166 Scuola di specializzazione in Pediatria. Università degli studi di Palermo, Palermo, Italy; 167 Neonatologia con UTIN e Nido. ARNAS Civico, Palermo, Italy; 168 Cystic Fibrosis Center, Department of Translational Medical Sciences University of Naples “Federico II”, Naples, Italy; 169 Centro Regionale Fibrosi Cistica, AOR Ospedale San Carlo, Potenza, Italy; 170 U.O. Pediatria II per la FIBROSI CISTICA (CRR) e le Malattie Respiratorie – Allergologia– ISMEP, Palermo, Italy; 171 Dipartimento di Scienze per la promozione della salute materno infantile G. D’Alessandro. Università degli studi di Palermo, Palermo, Italy; 172 U.O.S.D. Genetica molecolare. ARNAS Civico, Palermo, Italy

## S1 Effects of diet on cystic fibrosis microbiome

### Annamaria Bevivino (annamaria.bevivino@enea.it)

#### ENEA Casaccia Research Center, Sustainable Territorial and Production Systems Department, Rome, Italy

The human body contains complex communities of microorganisms that play an important role in human health. In the last decade, next-generation sequencing (NGS) was employed to decipher the structure and composition of the human microbiome, defined as the totality of microbes, their genomes and interactions in a defined environment. Cystic Fibrosis (CF) disease affects the function of a number of organs, principally the lungs, but also the gastrointestinal tract [1]. An increasing number of studies have revealed a cross-talk between the two compartiments as exemplified by intestinal complications during respiratory disease and *vice versa*. Although so far mechanisms through which the lung could influence the gut environment are unclear, the existence of the gut–lung axis could open up new possibilities for therapeutic approaches to respiratory diseases. A growing body of evidence suggests that the gastrointestinal (GI) microbiome in people with CF is altered. These dysbioses contribute to disease manifestations in many organs, both within and beyond the GI tract [2]. In this lecture, I highlight how changes in the intestinal microenvironment, with a particular focus on the intestinal microbiota, impact upon respiratory disease. Together with reported benefits for respiratory exacerbations, such as a reduced rate of pulmonary exacerbations and upper respiratory tract infections in patients with CF, the administration of probiotcs may globally modify the GI microbiome, thus promoting bacterial communities that modulate host immune responses [3]. Observations from clinical trials of probiotics in CF patient revealed the probiotics can ameliorate the dysbiosis of CF, reducing the proteobacterial populations in the gut microbiota, as well as decreasing gut inflammation and the number of pulmonary exacerbations.

**References**

1. Burke DG, Fouhy F, Harrison MJ, et al. The altered gut microbiota in adults with cystic fibrosis. BMC Microbiology. 2017;17:58.

2. He Y, Wen Q, Yao F, et al. Gut–lung axis: The microbial contributions and clinical implications. Critical Reviews in Microbiology. 2017; 43:81-95.

3. Van Biervliet S, Declercq D, Somerse S. Clinical effects of probiotics in cystic fibrosis patients: A systematic review. Clinical Nutrition ESPEN 2017;18: 37-43.

## S2 New technologies, new opportunities, new interpretative challenges in molecular genetics of Cystic Fibrosis

### Alessandra Coiana (acoiana@medicina.unica.it)

#### Dipartimento di Scienze Mediche e Sanità Pubblica, Università di Cagliari, Cagliari, Italy

Cystic Fibrosis (CF) occurs most frequently in caucasian populations. Although less common, this disorder have been reported in all the ethnicities. Currently, there are more than 2000 described sequence variations in CFTR gene, uniformly distributed and including variants pathogenic and benign (CFTR1:www.genet.sickkids.on.ca/). To date, only a subset have been firmily established as variants annotated as disease-causing (CFTR2: www.cftr2.org). The spectrum and the frequency of individual CFTR variants, however, vary among specific ethnic groups and geographic areas.

Genetic screening for CF with standard panels of CFTR mutations is widely used for the diagnosis of CF in newborns and symptomatic patients, and to diagnose CF carrier status. These screening panels have an high diagnostic sensitivity (around 85%) for CFTR mutations in caucasians populations but very low for non caucasians.

Developed in the last decade, Next-Generation Sequencing (NGS) has been the last breakthrough technology in genetic studies with a substantial reduction in cost per sequenced base and a considerable enhancement of the sequence generation capabilities. Extended CFTR gene sequencing in NGS includes all the coding regions, the splicing sites and their flankig intronic regions, deep intronic regions where are localized known mutations, the promoter and the 5'-3' UTR regions. NGS allows the analysis of many samples concurrently in a shorter period of time compared to Sanger method . Moreover, NGS platforms are able to identify CFTR copy number variation (CNVs), not detected by Sanger sequencing.

This technology has provided new and reliable approaches to molecular diagnosis of CF and CFTR-Related Disorders. It also allows to improve the diagnostic sensitivity of newborn and carrier screening molecular tests. In fact, bioinformatics tools suitable for all the NGS platforms can filter data generated from the gene sequencing, and analyze only mutations with well-established disease liability. This approach allows the development of targeted mutations panels with a higher number of frequent CF mutations for the target population compared to the standard panels and a consequent enhancement of the diagnostic sensitivity.

Moreover, in the emerging challenge of diagnosing CF in non caucasians patients, the possibility of customize a NGS targeted mutations panel should increase the diagnostic sensitivity when the target population has different ethnicities.

## S3 Update on Urinary incontinence in Cystic Fibrosis

### Annalisa Fogazzi, Fabiana Timelli, Sandra Signorini

#### Cystic Fibrosis Support Centre, Department of Paediatric Children’s Hospital, ASST- Spedali Civili, Brescia, Italy

##### **Correspondence:** Annalisa Fogazzi (annalisafogazzi@tiscali.it)

**Background**

The life expectancy of patient with Cystic Fibrosis (CF) is increased in the last forty years. As result, extrapulmonary complication increased such as urinary incontinence (UI). The UI in patient with CF is probably connected with the increasing of pressure on the pelvic floor during coughing. This report sums up published articles concerning the prevalence, severity, impact and management of UI, in patients with CF.

**Materials and methods**

References were identified searching Medline, Embase and PubMed using the medical subject headings ‘cystic fibrosis’ AND ‘urinary incontinence’. Articles concerning UI prevalence, impact on quality of life and treatment were included.

**Results**

The UI’s prevalence ranged from 30-69% for women and 19-49% for girls. In adult men, UI’s prevalence ranges from 5% to 15% [1,2]. UI has a negative effect on the performance of airway clearance, exercise and/or spirometry, and has variable impact on patients’ lifestyle. There is limited evidence about the prevention and treatment of UI in CF, but the pelvic floor exercises are effective at reducing leakage in the short time [3]. Optimal positioning and posture advice during cough and other airway clearance techniques should be given as a preventative measure [4].

**Conclusions**

In CF, UI is common and can interfere with respiratory care, daily activities and quality of life. In CF literature, there aren’t evidences of routine assessment of UI or approaches standardization to identifying UI between different CF centres [5]. In our clinical experience patients do not report UI presence and their discomfort. In literature the main barriers to discussion are embarrassment and lack of knowledge of UI treatment’s option. A simple booklet about UI could be an educational resource to build more awareness in women and girls affected by CF [6].

**References**

1. White D, Stiller K, Roney F. The prevalence and severity of symptoms of incontinence in adult cystic fibrosis patients. Physiother. Theory Pract. 2000; 16: 35–42.
2. Burge AT, Holland AE, Sherburn M, et al. Prevalence and impact of urinary incontinence in men with cystic fibrosis. Physiotherapy. 2015; 101: 166–70.
3. McVean RJ, Orr A, Webb AK, et al. Treatment of urinary incontinence in cystic fibrosis. J Cyst Fibros. 2003; 2:171–6.
4. Nankivell G, Caldwell P, Follett J. Urinary incontinence in adolescent females with cystic fibrosis. Paediatr Respir Rev. 2010 ;11:95-9.
5. Frayman KB, Kazmerski TM, Sawyer SM. A systematic review of the prevalence and impact of urinary incontinence in cystic fibrosis. Respirology. 2017 doi: 10.1111/resp.13125.
6. Tomezzoli S, Longhini B, Tartali C, et al. Urinary incontinence: a booklet as an informative tool for women with cystic fibrosis. Pediatr Pulmonol. 2016; 51: 481.

## S4 Update on the revision of consensus document about the genetic analysis in cystic fibrosis

### Marco Lucarelli (marco.lucarelli@uniroma1.it)

#### Dept. of Cellular Biotechnologies and Hematology; Italian Pasteur Institute, Cenci Bolognetti Foundation, Sapienza University, Rome, Italy

On behalf of SIFC working group for the revision of *Consensus* document about genetic analysis in cystic fibrosis.

**Background**

Genetic analysis of cystic fibrosis transmembrane conductance regulator (CFTR) gene is fundamental for diagnosis, prognosis and personalized therapy of cystic fibrosis (CF). Despite CF is a monogenic disease, its genetics is not easy. Consequently, the genetic analysis of CFTR gene should take under account a lot of different situations that should be specifically handled.

**Materials and methods**

According to current definitions, the revision underway can be defined as a *Consensus* document. A group of 23 experts of SIFC composes the “working group for the revision of *Consensus* document about genetic analysis in CF”. A list of the subjects to be treated was made by the working group. A first elaboration of each subject was done by small subgroups. The overall document was revised several times by the working group, harmonized and approved as a first draft. This draft is in submission for a first evaluation and revision to the board of SIFC and of the other scientific societies involved in the management of CF. After this revision, a further elaboration by the working group will be done, aimed to the inclusion of suggestions and to the production of a second draft to be finally approved by SIFC and other scientific societies.

**Results**

Three basic items of the *Consensus* document were identified: main text, risk tables and flowcharts. The first draft of the main text is ready. Risk tables and flowcharts will be added in the next step. The main text is composed of 6 preliminary sections: list of components of the working group; premise; abbreviations; definitions; abstract; introduction. Then, there are 7 general chapters: general description of the disease; genetic counselling; general strategies of mutational search (with appropriateness); mutational search in CF; mutational search in CFTR-related disorders; preimplantation mutational search; characterization of novel, rare and uncharacterized sequence variations. The next chapter describes specific strategies of mutational search and is divided into 13 sections each concerning mutational search applied to specific practical cases. The document finishes with the following 4 chapters: quality assurance; report; databases, website and software; references.

**Conclusions**

The need of documents assuring a homogenous behavior of geneticists and molecular biologists performing the CFTR genetic test is particularly felt. The last update of this type of document in Italy was done in 2005. The revised *Consensus* document will be an updated valuable practical guide, based on solid scientific evidences, for CF healthcare professionals.

## S5 Old and new tools in diagnostic microbiology

### Patrizia Morelli (patriziamorelli@gaslini.org)

#### Laboratory Microbiology CF- Giannina Gaslini Institute- Genoa, Italy

The main aim of clinical microbiology is the identification of bacterial, viral, fungal, and parasitic agents that cause human diseases, to enable an optimized clinical management of patients as well as to prevent infectious diseases transmission. In the last years the clinical microbiology has changed rapidly: in addition to culture-based methods that remain irreplaceable in routine clinical microbiology, news tools have been implemented, providing improved sampling and culture strategies and culture-independent methods. The inclusion of matrix-assisted laser desorption/ionization–time-of-flight mass spectrometry (MALDI-TOF MS) as diagnostic tool has improved the accuracy of pathogen identification and shortened the time to achieve the final results. The technological advances in non-cultured methods have unveiled a much larger human-associated microbiota than was expected. The airway microbiome has been found to be much more diverse than previously thought based on bacterial culture, and relationship between that diversity and pulmonary function has been identified. This review summarizes the state of the art of culture methods and molecular techniques in clinical microbiology laboratory. The most advanced level of newly developed techniques includes disease-based sampling kits, new culture approaches, direct pathogen detection, point-of-care (POC) testing and clinical isolate identification with MALDI-TOF MS. Despite the advent of cutting edge molecular-based (real time PCR, 16S rRNA sequencing, next-generation sequencing) and protein-based microbial identification tools, there is an enormous need to continue culture-based testing to assess susceptibility to all antimicrobials and to identify pathogens with mutations that may escape detection by new technologies. Rapid detection and identification of infectious agents in clinical specimens are mandatory to implement appropriate therapeutic measures. For this purpose it is clear that combining culture based methods and molecular techniques can contribute to clinical management of airway infection, highlighting the importance of making these two methods complementary.

## S6 Growth in Italy in the first two years of life. Data from the Italian Cystic Fibrosis Registry

### Rita Padoan^1^, Barbara Giordani^2^, Annalisa Amato^2^, Fabio Majo^3^, Gianluca Ferrari^4^, Serena Quattrucci^5^, Laura Minicucci^6^, Giovanna Floridia^7^, Gianna Puppo Fornaro^2^, Domenica Taruscio^4^, Marco Salvatore^4^

#### ^1^Centro Regionale di Supporto per la Fibrosi Cistica, ASST Spedali Civili di Brescia, Italy; ^2^Lega Italiana Fibrosi Cistica ONLUS, Roma, Italy; ^3^Unità Operativa Complessa Fibrosi Cistica Ospedale Pediatrico Bambino Gesù, Roma, Italy; ^4^Centro Nazionale Malattie Rare, Istituto Superiore di Sanità, Roma, Italy; ^5^Centro di Riferimento per la Fibrosi Cistica – Regione Lazio, Roma, Italy; ^6^Centro di Riferimento per la Fibrosi Cistica – Regione Liguria, Genova, Italy; ^7^Pre-BIO-Unità di bioetica, Istituto Superiore di Sanità, Roma, Italy

##### **Correspondence:** Rita Padoan (rita.padoan@asst-spedalicivili.it)

**Background**

Cystic fibrosis (CF) is a progressive genetic disease that affects multiple organ systems. Therapy is directed to maintain and optimize nutritional status and pulmonary function, as these are prognostic factors for maintaining good lung function during the pediatric-adolescent age and key factors in survival. For infants and young children, the aim is to achieve the 50th percentile of weight and length for a healthy same-age population up to age 2 years.CF diagnosis by means of neonatal screening, ever more precocious, offers the great opportunity to correct pancreatic insufficiency since its inception, thus enabling the correct therapy to achieve a good nutritional status for all children. However, as shown by studies in different CF population, this target is not achieved for all subjects. The purpose of this report is to present the growth data of children aged up to 24 months enrolled in the Italian Cystic Fibrosis Registry, in the years 2011-2014.

**Materials and methods**

Registry data of the period 2011-2014 were evaluated. Indicators used to assess growth and nutritional status were weight/length (W/L), and length per age. Data were analyzed by age (groups: 0-6 months, 7-12, 13-18, 19-24) and for each year of data collection. The indicators were standardized with the calculation of the z-score.

**Results**

The Table 1 presents the data (median z-score for length per age and percentage of infants with W/L z-score >50°centile) for the years 2011-2014. With regard to weight per length, the lowest median z-scores are found each year in the 0-6m age group. Recovery takes place within the first 12 months of life in most patients: for all years of study, the median z-score has passed from a negative value ​​in 0-6m group, to values around zero in the subsequent age groups, with progressive increase of children with parameters above 50 ° centile and progressive reduction of children below 10 ° centile.

**Conclusions**

Children with CF are born smaller than the average [1] and can already present at the time of diagnosis pancreatic insufficiency that has already impaired their growth. The six month figure therefore reflects the short period of diagnosis and the beginning of appropriate therapy. Over the period considered, at the age of 19-24 months, more than half of the infants have a W/L z-score above the 50°centile.

**Acknowledgements**

We would like to thank Italian CF Centers, which provide data to ICFR

**References**

1. Festini F, Taccetti G, Repetto T, et al. Gestational and neonatal characteristics of children with cystic fibrosis: a cohort study. J Pediatr. 2005;147:316-20.

**Table 1:** **(abstract M1).** Median z-score for length per age and percentage of infants with W/L z-score >50°centile in different age groups in 2011-2014 years

*Median z-score length/age*				
IRCF years	Age: 0-6 mo	Age 7-12mo	Age 13-18 mo	Age 19-24mo
2011	-0.5	0	0.2	0.1
2012	-0.75	-0.3	0.05	0.2
2013	-1	-0.1	0.25	-0.25
2014	-0.5	0.2	0	0.3
*Percentage of infants with a W/L z-score > 50° centile*				
2011	33	52	59	58
2012	27	30	52	54
2013	27	48	58	32
2014	33	54	51	80

## S7 Quality control of CFTR molecular analysis: experiences in Italy and Europe

### Manuela Seia (m.seia@policlinico.mi.it)

#### Medical genetics laboratory, Fondazione IRCCS Ca’ Granda Ospedale Maggiore Policlinico, Milan, Italy

The increasing interest in molecular biology applied to diagnostics is the result of the deep advances in scientific knowledge in genetics made possible by the introduction and the continuous development of applied technologies. The number of laboratories using molecular biology techniques is growing rapidly and the inevitable consequence of this "demographic explosion" is the need for standardization as well as a definition of the organization of laboratories. Guidelines recommend the participation of laboratories performing genetic testing in internal Quality Control processes, as well as in Quality Control Programs (CEQs).

The importance of laboratory participation in CEQ programs is recognized internationally.

Currently, quality control programs for the molecular analysis of the CFTR gene are available both at the Italian and the European level. In Italy the document of the State Region Conference of July 15, 2004 established that the Institute of National Public Health (Istituto Superiore di Sanità ISS) as the competent office to evaluate the technical and professional quality of the operators through external quality controls. Participation is open to both private and public laboratories.

In 2016, ISS examined 62 laboratories, each having received four DNA aliquots together with clinical and technical information. The laboratories sent back through a computerized system raw data and reports, written according to their standard format. Raw data and references were then evaluated by experts and a performance rating assigned: sufficient or insufficient.

The evaluation was done following the general evaluation criteria on the CNMR website at http://www.iss.it/tege/index.php.

An analogous scheme is proposed at European level by the European Cystic Fibrosis Thematic Network, which is a project approved under the 5th framework program of the European Union. This project is coordinated by the Human Genetic Center of the University of Leuven. Currently, CF Network collaborates with EuroGentest's European Molecular Genetics Quality (EMQN) in order to improve the harmonization of external quality evaluation systems in Europe.

At the moment the CF network, in order to improve harmonization of External Quality Assessment (EQA) schemes within Europe, works in close collaboration with the EuroGentest Network of Excellence and the European Molecular Genetics Quality Network (EMQN).

The European Network organizes an External Quality Assessment scheme for Cystic Fibrosis, for more than 200 laboratories world-wide, some performing diagnostic testing for cystic fibrosis, others working inside diagnostic kit companies.

The aim of the CF EQA scheme is to evaluate the entire analytical process, from DNA sample receipt and genotyping up to the written report with the final interpretation of the data as it is normally being sent to the clinician who requested for the genetic test.

Over the years, there has been a continuous improvement in performance both in Italy and in Europe, in accordance with the aims proposed by CEQ programs for educational and continuous improvement of quality.

## O1 A gene targeting approach for cystic fibrosis

### Silvia Pierandrei^1,2^, Giovanna Blaconà^1^, Valentina Salvati^3^, Giovanni Sette^3^, Giuseppe Cimino^4^, Federica Sangiuolo^5^, Adriana Eramo^3^, Marco Lucarelli^1,6^

#### ^1^Dept. of Cellular Biotechnologies and Hematology, Sapienza University of Rome, Rome, Italy; ^2^Dept. of Pediatrics and Child Neuropsychiatry, Sapienza University of Rome, Rome, Italy; ^3^Dept. of Oncology and Molecular Medicine, Italian Institute of Health, Rome, Italy; ^4^Regional Reference Center for Cystic Fibrosis, Umberto I Hospital, Rome, Italy; ^5^Dept. of Biomedicine and Prevention, Tor Vergata University of Rome, Rome, Italy; ^6^Italian Pasteur Institute, Cenci Bolognetti Foundation, Sapienza University of Rome, Rome, Italy

##### **Correspondence:** Silvia Pierandrei (lvia.pierandrei@uniroma1.it)

**Background**

Homologous replacement can be used to modify specific gene sequences of chromosomal DNA in a process called “Small Fragment Homologous Replacement” (SFHR). A wild-type small DNA fragment (SDF) is used to correct the mutated genomic target. Cellular responses and molecular mechanisms involved in SFHR are only partially understood. Different DNA repair pathways and cell cycle checkpoints, as well as specific genes (as for example Trex1), appear to mediate the cellular response. This approach can be used as therapeutic CFTR correction aimed at restoring the wild-type CFTR sequence. Recent studies have shown the possibility to expand patient-derived epithelial cells from different human tissues, through the “Culture Reprogramming Condition” (CRC) methodology. The possibility to obtain with high efficiency large amounts of primary epithelial cells with stem-cell properties from CF patients with different mutations would represent a pivotal advancement for CF gene targeting.

**Materials and methods**

To optimize the efficiency of SFHR, we developed a reporter based assay system where the replacement frequency is quantified by cytofluorimetric analysis following restoration of a stably integrated mutated eGFP gene in the genome of SV-40 immortalized mouse embryonic fibroblasts (MEF). Electroporation was performed using both Amaxa Nucleofection System (Lonza) and Attractene (Qiagen) reagent. For the quantitative expression analysis of involved genes, arrayed real-time PCR was performed. CRC technology consists of co-culture of primary epithelial cells with irradiated mouse fibroblasts as feeder layer, in the presence of the Rock inhibitor Y-27632. A preliminary setup and characterization of CF-CRC cells, obtained, after consent, from nasal brushing of CF patients, were performed.

**Results**

Amaxa Nucleofection System showed higher transfection efficiency than the Attactene reagent, respectively 76% and 25%. Both transfection systems presented high cellular toxicity, enhanced by the SDF. Best correction conditions in MEF consisted in G2/M phase cell cycle synchronization associated with hypomethylating treatment and low Trex1 gene expression. These conditions correlate with 0.071% of eGFP correction. CF-CRC cells resulted to express typical stem-like cell markers and retained the ability of re-differentiate to respiratory epithelium. Their CFTR mRNA resulted physiologically expressed and its sequence, as well as that of CFTR genomic DNA, resulted unmodified in respect to that of corresponding patient.

**Conclusions**

SFHR manipulations and molecular targets useful for increasing the replacement efficiency have been evidenced. Modulation of overall pathways and specific genes (for example Trex1) are suitable for a better comprehension of SFHR mechanism. The CRC technology applied to CF patient-derived cells may expand the supply of functional CF respiratory stem-like and differentiated cells for testing personalized therapeutic strategies, including drug testing and cellular therapy approaches.

## O2 Ivacaftor: clinical effects after two years of monitoring

### Mirella Collura^1^, Elisa Parisi^2^, Annalisa Ferlisi^1^, Gabriella Traverso^1^, Marcella Bertolino^1^, Lisa Termini^1^, Maria A Orlando^1^, Caterina Di Girgenti^3^, Valeria Pavone^2^, Maria A Calamia^1^, Maria G Silvestro^1^, Caterina Lo Piparo^1^, and Francesca Ficili^1*^

#### ^1^U.O. Pediatria II per la FIBROSI CISTICA (CRR) e le Malattie Respiratorie. ISMEP – Palermo, Italy; ^2^ Dipartimento di Scienze per la promozione della salute materno infantile G. D’Alessandro. Università degli studi di Palermo, Palermo, Italy; U.O.S.D. Genetica molecolare, ARNAS Civico, Palermo, Italy

##### **Correspondence:** Francesca Ficili (fficili@hotmail.com)

**Background**

Cystic fibrosis (CF) is the most common genetically determined, life-limiting disorder in populations of European. The genetic origin of CF is the mutations in the CF transmembrane conductance regulator (CFTR). CFTR mutations may be classified into 6 different categories based on the mechanisms that are affected. Class I results from mutations leading to absent CFTR production. Class II, including Phe508del, are caused by defective CFTR processing. Class III mutations result in expression of CFTR, the channel gating is defective and results in impaired chloride transport function. Conductance defects are seen in class IV mutation. Class IV result in a milder phenotype. Finally, class VI mutations are characterized by a functional but unstable CFTR. Ivacaftor is a potentiator that augmented chloride transport and increased airway surface liquid height and cilia beat frequency in airway epithelial cells expressing a CFTR gating mutation (III Class).

**Materials and methods**

Between September 2015 and September 2017 at the Regional Reference Center for the CF of Palermo, a total of 7 patients (female:4, male:3) aged 8–42 years (mean 25,6) with CF, carriers of a CFTR channel gating mutation, were enrolled and assigned to receive Ivacaftor (150 mg x2/die). We evaluated auxometry (weight, hight and BMI), respiratory function by spirometry (%FEV_1_ %FVC), liver, pancreatic and renal function, sweat test, respiratory infections and CF Questionnaire-Revised (CFQ-R). Nobody used to take CYP3A-inhibitors.

**Results**

We observed the improvement of respiratory function in term of FEV1 and FVC after only 3 months of observation. In fact the percent of FEV1 was +0.9% at T_3_, +3,6% after six months (T_6_) and +3,2% after two years (T_24_). FVC got better: + 3,4%, +3,18% and + 3,4% respectively at T_3_, T_12_, T_24_. The nutritional state was evaluated by the mean of Body Mass Index that increased of 2,02 points after 24 months of observation: from 21,28 Kg/m^2^ at T_0_ to 23,3 at T_24_ (Table 2). The health perception was investigated by the CFQ-R respiratory symptom scale: +14 points percent from T_0_ to T_24_. During this time we detected 10 pulmonary exacerbations. We didn't record any side effects, particularly monitoring liver function.

**Conclusions**

We are living a new era of precision medicine in CF. Kalydeco is an example of innovative therapeutic strategy for carriers of a CFTR channel gating mutation. We demonstrated the improvement of respiratory function in 24 months of clinical observation without side effects.

Further studies are needed to demonstrate the efficacy of this new drug in the clinical practice.

**Consent for publication:** The patients gave the consent to publish clinical data.

**Table 2:** **(abstract O2).** Evaluation of FEV 1, FVC, BMI, CFQR

	T_0_	T_3_	T_6_	T_12_	T_18_	T_24_
FEV1 (%)	74,8	75,7	78,4	78,8	77	78
FVC (%)	84,6	88	88,7	88,4	83	87
BMI (Kg/m^2^)	21,28	22,7	22,4	22,57	23,38	23,3
CFQR (%) (Respiratory)	81	-	87	89	-	95

## O3 Preliminary safety and efficacy of triple combination cftr modulator regimens in CF

### Carla Colombo^1^, Elizabeth Tullis^2^, Jane C Davies^3^, Charlotte McKee^4^, Cynthia DeSouza^4^, David Waltz^4^, Jessica Savage^4^, Marc Fisher^4^, Rebecca Shilling^4^, Sam Moskowitz^4^, Sarah Robertson^4^, Simon Tian^4^, Jennifer L Taylor-Cousar^5^, Steven M Rowe^6^

#### ^1^Fondazione IRCCS Ca' Granda, Ospedale Maggiore Policlinico, University of Milan, Milan, Italy; ^2^St Michael’s Hospital, Toronto, ON, Canada; ^3^Imperial College & Royal Brompton Hospital, London, UK; ^4^Vertex Pharmaceuticals Incorporated, Boston, MA, USA; ^5^National Jewish Health, Denver, CO, USA; ^6^University of Alabama at Birmingham, Birmingham, AL, USA

##### **Correspondence:** Carla Colombo (carla.colombo@unimi.it)

**Background**

Prior studies with CFTR modulators in pts heterozygous for *F508del* and a minimal function (MF) CFTR mutation (*F508del*/MF) have failed. One strategy to enhance clinical efficacy is to add a second corrector to established corrector/potentiator regimens. Safety and efficacy of 3 such next-generation (NG) correctors (VX-440, VX-152, VX-659) in triple-combination therapy (TC) with tezacaftor (TEZ) and ivacaftor (IVA) were evaluated in CF pts with *F508del*/MF or *F508del*/*F508del* genotypes.

**Materials and Methods**

Randomized, double-blind, placebo (PBO)- or active-controlled studies of VX-440 and VX-152 (Ph2), and VX-659 (Ph1) in TC were conducted in *F508del*/MF or *F508del/F508del* (VX-152 and VX-440 only; after 4 wks of TEZ/IVA pretreatment) CF pts. Primary objectives were safety and tolerability; efficacy was assessed by absolute change in ppFEV_1_ from baseline (BL) and pharmacodynamic effect by change in sweat chloride from BL.

**Results**

BL characteristics were balanced. NG TC regimens were well tolerated; most AEs were mild or moderate. AEs leading to discontinuation included increased ALT/AST (VX-440 TC, n=1), pneumonia (VX-152 TC, n=1), and respiration abnormal/sputum increased (PBO, n=1). After 2 to 4 wks, improvement in ppFEV_1_ of 9.6 to 12.0 percentage points was seen with all 3 NG TC regimens in *F508del*/MF pts, and 7.3 to 9.5 percentage points with VX-440 or VX-152 TC on top of TEZ/IVA in *F508del/F508del* pts (vs TEZ/IVA BL). Significant reductions in sweat chloride were also seen (Table 3).

**Conclusions**

This is the first demonstration of substantial improvements with NG TC regimens in *F508del*/MF CF pts, where previous CFTR modulators have failed, and in *F508del/F508del* CF pts. Ongoing phase 2 studies will guide development of future TC regimens.

**Table 3:** **(abstract O3).** Mean Absolute Change in ppFEV_1_ and Sweat Chloride (SwCl)

	*F508del/*MF			*F508del/F508del* ^*a*^ (all received active TEZ/IVA)		
	n	ppFEV_1_	SwCl (mmol/L)	n	ppFEV_1_	SwCl (mmol/L)
Through day 29^b^						
VX-440 (600 mg q12h)^c^ + TEZ/IVA^d^	18	+12.0*	-33.1*	20	+9.5*	-31.3*
Placebo	11	+1.4	+1.6	6	-2.5	+2.1
Day 15						
VX-152 (200 mg q12h)^c^ + TEZ/IVA^e^	10	+9.7^†^	-14.1^‡^	10	+7.3^‡^	-20.9^†^
Placebo	5	-0.9	+1.0	4	-1.4	+3.4
VX-659 (120 mg q12h)^c^ + TEZ/IVA^e^	9	+9.6^‡^	-41.6*			
Placebo	3	-0.4	-11.0			

^a^4-wk run-in period on TEZ/IVA. ^b^Average of days 15 and 29. ^c^Max dose tested. ^d^TEZ (50 mg q12h) + IVA (300 mg q12h). ^e^TEZ (100 mg qd) + IVA (150 mg q12h). *P<0.0001. ^†^*P*<0.01. ^‡^*P*<0.05. VX-440, least squares means; VX-152 and -659, observed means

## O4 Amniotic mesenchymal stem cells for cell therapy of Cystic Fibrosis: involvement of gap junctions in the rescue of CFTR-dependent chloride efflux

### Elisa Beccia^1,2^, Annalucia Carbone^1^, Maria Favia^3^, Stefano Castellani^1^, Manuela Seia^4^, Antonella Angiolillo^2^, Carla Colombo^5^, Valeria Casavola^3^, Massimo Conese^1*^

#### ^1^Department of Medical and Surgical Sciences, University of Foggia, Foggia, Italy; ^2^Department of Medicine and Health Sciences “V.Tiberio”, University of Molise, Campobasso, Italy; ^3^Department of Bioscience, Biotechnology and Biopharmaceutics, University of Bari, Bari, Italy; ^4^Medical Genetics Laboratory, Fondazione IRCCS Cà Granda Ospedale Maggiore Policlinico, Milan, Italy; ^5^Cystic Fibrosis Center, Fondazione IRCCS Cà Grande Ospedale Maggiore Policlinico, Department of Pathophysiology and Transplantation, University of Milan, Milan, Italy

##### **Correspondence:** Massimo Conese (massimo.conese@unifg.it)

**Background**

Stem cell-based therapy offers the opportunity to treating CF patients independently of mutation class. The amniotic membrane has been shown to be an ethical source for stem cells, since it is discarded after parturition. Human mesenchymal stem cells derived from the amniotic membrane (hAMSCs) display interesting properties for the purposes of treating CF lung disease, having the capacity to differentiate into epithelial cells, showing anti-bacterial activity and modulating the inflammatory process. We previously found that hAMSCs in co-culture with CF immortalized airway epithelial cells (CFBE41o- line, CFBE, homozygous for the F508del mutation) on Transwell filters acquired an epithelial phenotype and lead to the rescue of a mature and functional CFTR protein (Paracchini et al., J. J Biomed Biotechnol. 2012;2012:575471; Carbone et al., J Cell Mol Med. 2014;18:1631-43). The studies also highlighted that the rescue of CFTR was absent when separate co-cultures were obtained suggesting that cell-to-cell contacts are necessary for obtaining the F508del CFTR-dependent rescue.

**Materials and Methods**

In order to explore the role of gap junctions (GJs) in this rescue, co-cultures (hAMSC:CFBE, 1:5 ratio) were studied for the formation of GJs, before and after silencing them with siRNA directed against connexin 43 (Cx43), a major component of GJs. Under these experimental conditions, GJ-mediated intercellular communication was studied by the transfer of lucifer yellow, CFTR expression by confocal microscopy and Western blotting, while its activity by spectrofluorimetric measure of chloride efflux.

**Results**

Cx43 specific siRNA mediated down-regulation of Cx43 mRNA and protein, whereas a scrambled siRNA had no effect. Co-cultures could form functional GJs that were inhibited when the Cx43 protein expression was down-regulated. Transfection of co-cultures with siRNA against Cx43 resulted in the absence of CFTR expression on the apical membrane and reduction in the mature form of CFTR (band C), paralleled by the significant decrease in CFTR-dependent chloride channel activity.

**Conclusions**

These results indicate that GJs are involved in the correction of CFTR chloride channel activity upon the acquisition of an epithelial phenotype by hAMSCs in co-culture with CF cells. These results may indicate that undifferentiated hAMSCs may be administered to the CF lung where they would rescue CFTR expression and function by forming GJs. Further exploration of the usefulness of hAMSCs in the rescuing of CFTR-dependent basic defects involved in the pathogenesis of CF lung disease should be studied in primary airway epithelial cells obtained from CF patients and in "in vivo" models.

## O5 Cystic Fibrosis and meconium ileus: a multicentric study on risk factors for adverse outcome in infancy

### Rita Padoan^1*^, Bruno M Cesana^2^, Diego Falchetti^3^, Fiorella Battistini^4^, Elisabetta Bignamini^4^, Cesare Braggion^4^, Mirella Collura^4^, Natalia Cirilli^4^, Maria C Lucanto^4^, Vincenzina Lucidi^4^, Antonio Manca^4^, Valeria Raia^4^, Novella Rotolo^4^, Donatello Salvatore^4^, Sonia Volpi^4^

#### ^1^Centro Regionale di Supporto per la Fibrosi Cistica, Clinica Pediatrica, Ospedale dei Bambini, ASST Spedali Civili Brescia, Brescia, Italy; ^2^Dipartimento di Medicina molecolare e Traslazionale, Medicina e Chirurgia, Università di Brescia, Brescia, Italy; ^3^Chirurgia Pediatrica, H Niguarda Ca’ Granda, Milan, Italy; ^4^Centri Fibrosi Cistica di Brescia, Cesena, Torino, Firenze, Palermo, Ancona, Messina, Rome, Italy Bambino Gesù, Bari, Napoli, Catania, Potenza, Verona, Italy

##### **Correspondence:** Rita Padoan (rita.padoan@asst-spedalicivili.it)

**Background**

Meconium ileus (MI) is recognized as a risk factor for worse growth in early years in Cystic Fibrosis (CF) subjects, as demonstrated by a previous study in an Italian CF population aged less than 12 months (FFC project #19/2012). We do not yet have any epidemiological data or clinical information on early life and on clinical follow-up for Italian CF patients with MI. We suggest that risk factors associated to poor clinical outcomes might be identified in patients with MI, and eventually some of them might be modifiable. The first aim of the study is to identify risk factors associated to poor clinical outcomes in the first year of life in MI infants. Secondary aim is to describe complications presented in early age in this group of patients.

**Materials and methods**

Subjects with MI born in the years 2009-2015 and followed in 13 Italian CF Centers were enrolled. A database was built for the collection of data: diagnosis of ileus, surgical history, (medical or surgical MI, simple or complicated), age at CF diagnosis, medical and surgical treatments, anthropometric data and follow up from birth to 12 months. Adverse outcomes were considered the failure to grow and/or chronic Pseudomonas within the 1 year and death. Some variables evaluated as possible risk factors for an adverse outcome are: presence of surgical MI, complicated MI, presence of stoma, duration of the first hospitalization, parenteral nutrition, age at CF specialist visit.

**Results**

Eighty five subjects were enrolled from 13 centers. The study covers about 70% of Italian MI cases as reported by the Italian CF Registry data for 2015 year (85/121). 39 are males. In 20 subjects (24%) prenatal diagnosis of intestinal occlusion was posed and 11/20 (55%) presented with a concomitant ileal atresia. 71 (84%) were surgical MI, of which 33 (46%) were complicated. 40/71 (56%) were resected, and in 41 (58%) a stoma was packaged. Cholestasis was reported for 18/85 (21%). In 9 out of 50 (18%) IRT value at birth was negative. 55% (47 subjects) was breast-fed. Full analysis of risk factors will be presented.

**Conclusions**

Up to now there are no shared recommendations for the management of infants with MI among pediatric surgeries, neonatal units and CF centers. Appropriate improvement programs for CF diagnosis and follow up in the first year of life of MI infants are needed.

**Acknowledgements**

This study was conducted thanks to a grant from the Foundation for Research in Cystic Fibrosis (project #28/2015)

## O6 Relationship between glucose and insulin response during an oral glucose tolerance test (OGTT) and lung clearance index in cystic fibrosis patients

### Erica Nazzari^1^, Riccardo Guarise^1^, Palmiro Mileto^1^, Francesca Garbarino^1^, Gianfranco Alicandro^3^, Alberto Battezzati^2^, Carla Colombo^1^

#### ^1^Fondazione IRCCS Ca’ Granda Ospedale Maggiore Policlinico, Regional Cystic Fibrosis Center, Milan, Italy; ^2^ICANS, Università degli Studi di Milano, Milan, Italy; ^3^Dipartimento di Scienze Cliniche e di Comunità, Università degli Studi di Milano, Milan, Italy

##### **Correspondence:** Erica Nazzari (erica.nazzari@policlinico.mi.it)

**Background**

Impaired glucose tolerance and cystic fibrosis related diabetes (CFRD) negatively affect respiratory function measured by FEV1. Insulin secretory defects are risk factors for CFRD and may be also related to early lung function abnormalities that cannot be detected by spirometry. Our aim was to assess the correlation between OGTT-derived indices and the Lung Clearance Index (LCI), a more sensitive marker of early lung abnormalities than FEV1.

**Materials and methods**

We performed a Multiple-Breath Washout Test in 61 CF patients with a mean age of 18 years (range: 10-30) who underwent an OGTT as part of their annual screening for CFRD. From OGTT, we retrieved data on 2h plasma glucose (as a marker of glucose tolerance) and 30 min plasma insulin (as a marker of early insulin response). We compared LCI values across strata of glucose tolerance and quartiles of 30 min plasma insulin after OGTT. Moreover, we fitted multiple linear regression models to take into account the potential confounding effect of sex and age.

**Results**

According to the OGTT, 7 patients (11.5%) had diabetes without fasting hyperglycemia and 11 patients (18%) showed impaired glucose tolerance. Patients in the lower quartiles of 30 min plasma insulin after OGTT had increased LCI values as compared to the higher quartiles (median across quartiles: Q1: 14.5, Q2: 15.8, Q3: 12.7, Q4: 11.0, P= 0.029), while the LCI was not significant different (P= 0.30) among patients with diabetes (14.6, IQR: 12.1; 19.0), glucose intolerance (14.1, IQR: 13.0; 17.1) and patients with normal glucose tolerance (median: 13.4, IQR: 9.9; 16.4). In multiple linear regression models adjusted for sex and age, 2h plasma glucose (β= 0.03, P= 0.016) and 30 min plasma insulin (β= -0.04, P=0.01) were significant predictors of LCI.

**Conclusions**

In CF patients, hyperglycemia and reduced early insulin response are independently associated with early lung function abnormalities, as detected by LCI. Our results could help to clarify the role of insulin secretory defects in the deterioration of respiratory function in CF.

**Acknowledgements**

Supported by grants from Italian Cystic Fibrosis Research Foundation and from Regione Lombardia.

**Consent for publication:** Written informed consent was obtained from the patients and/or their parents for publishing clinical data.

## O7 Pregnancy in CF women: an observational study from the Italian Cystic Fibrosis Registry (ICFR)

### Barbara Giordani^1*^, Annalisa Amato^1^, Fabio Majo^2^, Gianluca Ferrari^3^, Serena Quattrucci^4^, Laura Minicucci^5^, Rita Padoan^6^, Giovanna Floridia^7^, Gianna Puppo Fornaro^1^, Domenica Taruscio^3^, Marco Salvatore^3^

#### ^1^Lega Italiana Fibrosi Cistica ONLUS, Rome, Italy; ^2^Unità Operativa Complessa Fibrosi Cistica Ospedale Pediatrico Bambino Gesù, Rome, Italy; ^3^Centro Nazionale Malattie Rare, Istituto Superiore di Sanità, Rome, Italy; ^4^Centro di Riferimento per la Fibrosi Cistica – Regione Lazio, Rome, Italy; ^5^Centro di Riferimento per la Fibrosi Cistica – Regione Liguria, Genova, Italy; ^6^Centro di Supporto per la Fibrosi Cistica – Regione Lombardia, Brescia, Italy; ^7^Pre-BIO-Unità di bioetica, Istituto Superiore di Sanità, Rome, Italy

##### **Correspondence:** Barbara Giordani (gonexa@gmail.com)

**Background**

Scientific literature about pregnancies in women with a diagnosis of cystic fibrosis (CF) is still scattered. Family planning in this group of women is becoming commoner with health improvement and survival increase. The objective of the study is to assess the maternal health status of patients with cystic fibrosis through the analysis of maternal outcomes.

**Materials and methods**

We conducted a longitudinal analysis of functional outcomes in pregnant women over the period 2010-2015, using records from the Italian Cystic Fibrosis Registry (ICFR). Cases were matched by age and genotype with never pregnant control subjects (1:2). Characteristics at baseline were outlined in both comparison groups. Changes in lung function and nutritional outcome (BMI) were also assessed.

**Results**

A total of 84 pregnancies were identified in 81 women. Median maternal age was 31 years. Pancreatic insufficiency was reported in 50% of pregnant women. 55% presented a BMI <22, 44% had a moderate-severe respiratory function (FEV_1_% predicted <70%). Chronic *P.aeruginosa* was present in about half of cases. Evidence of difference in median age at CF diagnosis was found between pregnant and never pregnant women (P<0.05). A decrease in lung function was found statistically significant in pregnant women over the period 6-12 months before pregnancy and 12 months after delivery (P<0.5). No significant differences were found over the same study period between pregnant and the control group in nutritional status and lung function. Only one patient died 14 months after delivery.

**Conclusions**

This study showed a decline in lung function over the ante-post pregnancy period in patients with cystic fibrosis. However, further investigations are needed to improve knowledge about the long period impact of pregnancy on the cystic fibrosis disease.

We would like to thank Italian CF Centers, which provide data to ICFR

## O8 Cystic Fibrosis: the sense of smell

### Antonella M Di Lullo^2,4*^, Marika Comegna^1,2^, Felice Amato^1,2^, Paola Iacotucci^3^, Vincenzo Carnovale^3^, Elena Cantone^4^, Maurizio Iengo^4^, Giuseppe Castaldo^1,2^

#### ^1^Department of Molecular Medicine and Biotechnology, University of Naples Federico II, Naples, Italy; ^2^CEINGE- Advanced Biotechnology, Naples, Italy; ^3^Regional Center of Cystic Fibrosis, Adult Section, Department of Medical Traslational Science, University of Naples Federico II, Naples, Italy; ^4^Department of Neuroscience, ENT Section, University of Naples Federico II, Naples, Italy

##### **Correspondence:** Antonella M Di Lullo (antonella.dilullo@libero.it)

**Background**

Cystic Fibrosis (CF) consists of multiorgan manifestations that include chronic rhinosinusitis (CRS) with or without nasal polyposis. The principal symptoms of CRS are nasal congestion, rhinorrea, mouth breathing, facial pain, and olfactory dysfunction (anosmia/hyposmia). A large percentage of CF patients present CRS, therefore we evaluated the olfactory performance in: i) CF patients with CRS without polyps; ii) age-matched healthy controls.

**Materials and methods**

We enrolled:

- group I: 29 CF patients with CRS without polyps with different mutations (mean age: 30.1±9.3 years);
- group II: 29 age-matched healthy volunteers (mean age: 29.7±5.9 years).

All cases were not smokers. They did not assume food or beverages other than water within 6 hours previous test. Subjects with acute nasal infection, facial trauma and/or nasal polyps were excluded. All subjects underwent the Sniffin’Sticks (Burghart Medical Technology) (SS) to assess the olfactory performance.

The study obtained the approval of local ethical committee.

**Results**

Group I: 7% of CF patients were normosmic, 69% hyposmic, 10% anosmic, 14% borderline. They presented mean odor identification of 10.55±2.99, mean odor discrimination of 10.28±2.67, mean odor threshold of 2.14±2.63, mean TDI score of 22.97±6.07. All these olfactory evaluations were statistically different to healthy controls (Group II) (p<0.001).

**Conclusions**

The impairment of olfaction influences the quality of life in CF patients, decreasing appetite, aggravating nutritional problems and playing a significant role in social interactions. We found a significant frequency of smelling disorders in CF patients and a major impairment of odor threshold. Our data suggest that CF olfactory impairment can result from olfactory periphery dysfunction due to either conduction problems by CRS and impaired mucociliary clearance owing to thickened mucus. So therapies improving the mucociliary clearance might recover olfactory performance in CF patients with CRS. Moreover this olfactory test could represent a new clinical tool in the follow-up of CF patients.

**Acknowledgements**

We acknowledge Ministero della Salute (Rome, Italy) L.548/93 for the regional research funding quote of years 2007-12.

## O9 Bronchoscopy as a therapeutic approach in Cystic Fibrosis

### Claudio Orlando^1^, Alida Casale^2^, Angela Sepe^2^, Fabiola De Gregorio^2^, Antonia De Matteo^2^, Alice Castaldo^2^, Chiara Cimbalo^2^, Antonella Tosco^2^, Valeria Raia^2^

#### ^1^Department of “Endoscopia Respiratoria Pediatrica e Vie Aeree Difficili”, AORN Santobono-Pausilipon, Naples, Italy; ^2^Cystic Fibrosis Center, Department of Translational Medical Sciences University of Naples “Federico II”, Naples, Italy

##### **Correspondence:** Claudio Orlando (claendo@gmail.com)

**Background**

Atelectasis is a common complication in cystic fibrosis (CF) patients. Several patients do not respond to standardized therapies. As persistence of atelectasis is associated with poor outcome, bronchoscopy can represent an alternative tool for the treatment of this complication. Few data are available for its routine use. Our objective was to evaluate the efficacy of bronchoscopy as a therapeutic tool in CF pediatric patients with atelectasis/subatelectasis not responding to conventional therapies.

**Materials and methods**

Since 2013 10 CF patients with atelecatsis underwent one or more bronchoscopies. For each patient, microbiological and radiological data, respiratory function (FEV1% and MMEF%) were collected. Flexible Olympus Bronchoscopy was used. Broncho-alveolar-lavage was performed and mucolytics and antibiotics were instilled. In few occasions surfactant was employed. Following the procedure, patients underwent PEP-Mask respiratory physiotherapy/non-invasive ventilation in C-PAP mode; after 48-72h RX chest was performed. Subsequently, patients were regularly followed.

**Results**

Ten patients (4 males) were enrolled (mean age 9.6±4.7 years, 6 months-16 years); 9/10 had pancreatic insufficiency. Indication for 1st bronchoscopy was always the radiological finding of atelectasis/subatelectasis not responsive to conventional therapy. In 7/10, radiological examinations showed a single atelectasis (2/7 due to ABPA); in 3/10 multiple atelectasis. 7/10 and 3/10 underwent a single and repeated bronchoscopy (through a mean period of 6 months) respectively. 18 procedures were performed: dornase-alpha was always used and in 17/18 patients it was associated with 7% hypertonic solution and hyaluronic acid. In 15/18 procedures colistin (14/18) or tobramicin (1/18) was instilled in case of Pseudomonas aeruginosa infection, while in 2/18 vancomycin in presence of Staphylococcus aureus. In 2/18 cases, surfactant was used for the largest extension of the atelectasis area. In patients undergoing a single procedure, complete resolution of atelectasis was observed in 6/7, while in 1/7 resolution was partial. In 3/10 patients undergoing repeated bronchoscopy, complete resolution of atelectasis was observed in 1/3 after 3 procedures, in 2/3 X-ray showed a partial improvement. The mean values of FEV1% and MMEF% pre and post-bronchoscopy increased significantly (M±DS 74.69±12.44 vs 84.00±16.58; *p*<0.005; 40.62 ± 14.68 vs 55.23±23.01, *p*<0.005 respectively).

**Conclusions**

Bronchoscopy can play an important role in resolution of atelectasis. Our results show that the procedure is well tolerated and can lead to radiological resolution of atelectasis even after a single procedure. Clinical benefits were evident in terms of statistically significant improvement of spirometry parameters. A national longitudinal observational study could be useful to draw up a standardized protocol.

**Consent for publication:** Written informed consent was obtained from the patient’s parents.

## O10 Survival after lung transplantation for Cystic Fibrosis in Italy: a single center experience of 20 years follow-up

### Daniela Savi^1,2^,Serena Quattrucci^3^, Michela Mordenti^1^, Enea Bonci^4^, Patrizia Troiani^3^, Viviana D’Alù^1^, Paolo Rossi^3^, Monica Varchetta^3^, Tamara Perelli^3^, Serenella Bertasi^3^, Paolo Palange^1^, Giuseppe Cimino^1^

#### ^1^Department of Public Health and Infectious Diseases, Adult Cystic Fibrosis Center, “Sapienza” University of Rome, Rome, Italy; ^2^Cystic Fibrosis Unit, Bambino Gesù Children’s Hospital, Rome, Italy; ^3^Department of Pediatrics, Cystic Fibrosis Center, “Sapienza” University of Rome, Rome, Italy; ^4^Department of Sperimental Medicine, “Sapienza” University of Rome, Rome, Italy

##### **Correspondence:** Daniela Savi (danielasavi1@virgilio.it)

**Background**

Despite new medical therapies that have delayed the progression of lung disease with the consequent improvement of life expectancy, lung transplantation is currently the only treatment for end-stage respiratory insufficiency in patients with cystic fibrosis (CF). In this study we analyzed retrospectively our center’s experience since start of the transplantation program in 1996 with focus on survival analysis.

**Materials and Methods**

We reviewed CF transplant database and patient charts. Survival rates were calculated and compared between four era from 1996 to 2016. Categorical data were presented as number and percentage and comparisons done using the *chi-square (χ*^*2*^*) test* or *Fisher’s exact test*. Numeric data were presented as mean ± standard deviation (SD), for normally distributed data and comparisons done using the two sample independent *t-test*, while non-normal numeric data were presented as median (range).

**Results**

In a 20–year period, 243 patients with CF were listed for lung transplantation; 125 patients (62 males, 63 females) underwent transplantation and 85 patients died while waiting for donor organs. The mean and median age at transplantation was 27 ± 8 SD and 26 (range 9–52) years, respectively. FEV1 was 27.7 ± 6.3 % predicted, 115 patients (92 %) were pancreatic insufficient and 43 patients (34 %) had diabetes CF-related. Overall 1-year survival after lung transplantation for CF was 71.2 %, 5-year survival was 56.9 %, 10-years survival was 46.8 %, 15-year survival was 31.6 % and 20-years survival was 35%. Removing CF patients who died within the first three postoperative months, the mean and median survival after transplantation were 8 ± 5.6 SD years and 7.1 years (range 3 months – 20 years), respectively. We found a trend for gender difference in survival rate (CF males 7 ± 6.2 SD years *versus* CF females 5.4 ± 5.6 SD years, *p* < 0.07).

**Conclusions**

Lung transplantation is a well-established life extending treatment for patients with CF. Survival after lung transplantation in our centre is good, although slightly lower than the international data. Further analysis is needed to identify the multifactorial mechanisms that might have contributed to reduce outcomes at our center.

## O11 New therapeutic strategies for the treatment of allergic bronchopulmonary aspergillosis in patients with Cystic Fibrosis

### Lucia Tardino, Giuseppe F Parisi, Anna Portale, Chiara Franzonello, Maria Papale, Novella Rotolo, Salvatore Leonardi

#### U.O.C. Broncopneumologia Pediatrica e Fibrosi Cistica, University of Catania, Catania, Italy

##### **Correspondence:** Lucia Tardino (lucia-18@hotmail.it)

**Background**

Allergic bronchopulmonary aspergillosis (ABPA) is a pulmonary disorder, which occurs in patients with chronic lung diseases such as Cystic Fibrosis (CF). ABPA results from hypersensitivity to *Aspergillus* antigens which determines airways inflammation, bronchiectasis and bronchospasm. Conventional therapy consists of the use of steroids combined with anti-mycotic drugs. In conventional treatment-resistant cases, it is accredited therapy with Omalizumab, a recombinant humanized murine monoclonal antibody that binds to free IgEs, impinging on the receptors present on mastocytes and basophils. This article describes our experience on the use of anti-IgE monoclonal antibody in three CF patients with ABPA.

**Case Reports**

I, A 17-year-old male CF patient was admitted to our hospital for respiratory distress, wheezing and persistent cough. His sputum cultures were chronically positive for *S.aureus* and recently positive for *Aspergillus fumigatus (Af)*. He complained of persistent dry cough and his FEV1 has progressively reduced from 75% to 40% of predicted. Total IgE were 1324 UI/ml, specific IgE for *Af* levels were 23.9 kUA/l and IgG antibody to *Af* were positive, making possible the diagnosis of ABPA. Thus, we started treatment with Prednisone and Itraconazole. However, after several weeks his general conditions worsened. We decided to start treatment with Omalizumab according to standard protocols. Symptoms and FEV1 improved after the second administration. At week 8 of therapy, his FEV1 raised to 83% predicted. After three months IgG antibody to *Af* were negative and after one year total IgE were <363 UI/ml.

II, 11-year-old male CF patient was admitted due to chronic respiratory failure.His airways were chronically colonized from for *P.aeruginosa* and in the previous two months from *Af*. Targeted antibiotic therapy was administered without clinical improvement. Total IgE 988 UI/ml, positive skin prick tests, RAST and airways cultures for *Af*, were directed towards a diagnosis of ABPA. Despite of steroids and antifungal therapy, he did not improve. Therapy with Omalizumab was started with improvement (FEV1 increased of 25%).

III, 42-year-old male patient with late CF diagnosis and ABPA, total IgE 988 UI /ml, skin prick test, RAST and positive sputum cultures for *Af*. His general conditions were poor with persistent cough and impaired lung function. After conventional therapy, which did not lead to some improvement, Omalizumab was started also in this case with a FEV1 increase of 22%.

**Conclusion**

Our experience demonstrates that treatment with Omalizumab may definitely improve general conditions and lung function in CF patients with ABPA refractory to conventional therapy.

**Consent for publication:** All patients gave the consent to publish clinical data.

## P1 Plasmatic Aminoacids in Patients with Cystic Fibrosis: An Observational Study

### Giuseppe F Parisi, Lucia Tardino, Maria Papale, Chiara Franzonello, Francesca Pennisi, Novella Rotolo, Salvatore Leonardi

#### Department of Clinical and Experimental Medicine, University of Catania − Catania, Italy

##### **Correspondence:** Giuseppe F Parisi (giuseppeparisi88@hotmail.it)

**Background**

Malnutrition in patients with Cystic Fibrosis results from a mismatch between nutrient requirement and consumption. Energy deficit depends on 3 factors: lost energy, energy taken with food and energy expenditure. Genetic mutation depletes Cystic Fibrosis Trans-membrane. Regulator (CFTR) function on the surface of epithelial cells in the digestive tract and in other compartments, where Cl-, other ions and water secretions are impaired. This modifies pH and dehydrates secretions that precipitate and obstruct the lumen, causing inflammation and damages. Associated conditions include exocrine pancreatic insufficiency, impaired bicarbonate and bile secretion and aberrant mucus formation, leading to maldigestion and malabsorption, particularly of fats and fat-soluble vitamins. Multiple factors can contribute to the reduction in energy intakes such as anorexia, gastroesophageal reflux, Distal Intestinal Obstruction Syndrome and lung inflammation.

Declining pulmonary function is associated with the Resting Energy Expenditure (R.E.E.) increase from 10 to 20%. Chronic lung disease exacerbations lead to an increased R.E.E. value, which returns to basal levels some weeks after resolution of inflammation. Attempting to balance the energy gap justifies precocious and aggressive nutritional intervention, which begins in the early years and continues throughout life. However, an increase in caloric intake is not sufficient to neutralize protein-calorie need resulting from R.E.E. value growth. Non-energy intake results in reduced respiratory muscle function and decreased exercise tolerance, causing a chronic and irreversible deterioration in patient status, until death.

**Materials and methods**

The aim of our study is to observe and analyze the evolution of Plasmatic AminoAcids in a sample of 34 CF patients, 17 men and 17 women, treated with appropriate low-carb, high-fat, high-calorie, high-glucose diet, tailored to anthropometric values, age and gender, as well as recommended by the latest guidelines, and to assess a possible correlation with the patient’s clinical phenotype nutritional state.

**Results**

Aminoacidogram showed that: 16/34 patients (47%) had significantly reduced Plasmatic Amino Acid levels; when considering patients with severe malnutrition, 63% presented an altered Amino Acid profile, although 30% of those with good nutritional status also had lowered levels (Table 4). These results suggest the presence of a metabolic disorder, which does not depend solely on nutritional status.

**Conclusions**

In conclusion, the amino acid profile seems to be influenced by different factors and somehow identifies a “metabolic disorder” that characterizes CF. Furthermore it does not appear to be outweighed only through high-calorie diet. Future studies and larger clinical samples will be needed.

**Consent for publication:** The patients gave the consent to publish clinical data.

**Table 4:** **(abstract P1).** Amino acid profile in different CF phenotypes

		**Pancreatic Insufficiency**	**Pancreatic Sufficiency**	
Low AA levels		15 (62%)	1 (10%)	16
Normal AA levels		9	9	18
		24	10	34
P<0.01				
	**FEV1 <50%**	**50%<FEV1<70%**	**FEV1 >70%**	
Low AA levels	8 (89%)	5 (38%)	3 (25%)	16
Normal AA levels	1	8	9	18
	9	13	12	34
P<0.1				
	**BMI <17**	**18<BMI<19**	**BMI >19**	
Low AA levels	7 (63%)	3	6 (30%)	16
Normal AA levels	4	0	14	18
	11	3	20	34
P<0.05				
		**PI + FEV1 <50%**	**PS +FEV1>70%**	
Low AA levels		8 (89%)	1 (14%)	9
Normal AA levels		1	6	7
		9	7	16
P<0.01				

## P2 CFTR complex alleles: a literature review

### Sabina M Bruno^1^, Giulia Licciardello^1^, Silvia Pierandrei^1,2^, Giampiero Ferraguti^1^, Serena Quattrucci^3^, Marco Lucarelli^1,4^

#### ^1^Dept. of Cellular Biotechnologies and Hematology, Sapienza University of Rome, Rome, Italy; ^2^Dept. of Pediatrics and Child Neuropsychiatry, Sapienza University of Rome, Rome, Italy; ^3^Regional (Lazio) Reference Center for Cystic Fibrosis, Umberto I Hospital, Rome, Italy; ^4^Italian Pasteur Institute, Cenci Bolognetti Foundation, Sapienza University of Rome, Rome, Italy

##### **Correspondence:** Marco Lucarelli (marco.lucarelli@uniroma1.it)

**Background**

Cystic Fibrosis (CF) is characterized by a wide variability of clinical expression because of the large number of different mutations affecting the Cystic Fibrosis Transmembrane conductance Regulator (CFTR) gene as well as several other factors, such as complex alleles (with two or more mutations in *cis* on a single allele). A suitable knowledge of the relationship between the genotype and the phenotype in CF is based on a better understanding of the basic mechanisms that act on the pathway from the mutated genotype to residual functional protein and, finally, to the clinical phenotype. In turn, this knowledge will improve diagnostic and prognostic capabilities, as well as therapeutic strategies. In particular, the occurrence of complex alleles could explain a part of the clinical variability found in CF, since sequence variations in *cis* on the same allele often affect transcript levels and/or the residual functionality of the CFTR protein. The presence of unidentified complex allele in a group of patients with the same apparent mutations, but different clinical manifestations, could explain the different severity of their conditions.

**Materials and methods**

In order to collect the currently available information on CFTR complex alleles, a thorough bibliographic research has been conducted. Overall, 130 papers were evaluated and all the information was collected in a summary table including the list of CFTR complex alleles currently known.

**Results**

About one hundred complex alleles were listed. For each complex allele one or more bibliographic sources, the occurrence in CF or CFTR-RD patients and, whenever possible, the differential effect at clinical and cellular level (compared to the effect of single variations), as well as any confirmatory studies, were reported.

**Conclusions**

Only for a small part of the CFTR complex alleles already known the mechanism of action and the pathogenic role has been clarified. Functional studies are mandatory to understand the role of the identified complex alleles in the different clinical manifestations of disease. The knowledge of the pathogenic effect of the complex alleles may improve diagnosis and prognosis of the disease and is expected to be crucial both to define the individual outcome and to guide the choice of the most suitable therapeutic approach for each patient.

## P3 Haplotypes possibly modulating CFTR expression

### Giampiero Ferraguti^1^, Manuela Sterrantino^1^, Sabina M Bruno^1^, Silvia Pierandrei^1,2^, Giancarlo Testino^1^, Giuseppe Cimino^3^, Serenella Bertasi^3^, Marco Lucarelli^1,4^

#### ^1^Dept. of Cellular Biotechnologies and Hematology, Sapienza University of Rome, Rome, Italy; ^2^Dept. of Pediatrics and Child Neuropsychiatry, Sapienza University of Rome, Rome, Italy; ^3^Regional (Lazio) Reference Center for Cystic Fibrosis, Umberto I Hospital, Rome, Italy; ^4^Italian Pasteur Institute, Cenci Bolognetti Foundation, Sapienza University of Rome, Rome, Italy

##### **Correspondence:** Giampiero Ferraguti (giampiero.ferraguti@uniroma1.it)

**Background**

The correlation between sequence variations of CFTR (Cystic Fibrosis Transmembrane conductance Regulator) gene (genotype) and clinical manifestations (phenotype) of Cystic Fibrosis (CF) is still difficult to understand completely. CFTR gene produces a primary transcript of 6132 bases; a complex transcriptional regulation occurs on the entire locus of the gene, with a dynamic not yet completely clear. In order to study the possible mechanisms involved in the modulation of these processes, we have conceived a study that analyzes selected intragenic haplotypes, possibly modulating CFTR expression, in different populations of CF and CF-like patients.

**Materials and Methods**

From a previous work, we selected eight markers (six SNPs and two polymorphic traits), that we analyzed through DNA sequencing of the CFTR gene in the following seven different populations: men with idiopatic seminal hyperviscosity (ISHV) and with congenital bilateral absence of vas deferens (CBAVD), patients with CFTR related disorders (CFTR-RD), CF patients with or without pancreatic sufficiency (CF-PS and CF-PI) as target populations; general population (GC) and normospermic men (NC) as controls. We obtained RNA samples from nasal brushing of selected patients, previously genotyped. RNA was analyzed by reverse transcriptase PCR assays to study a possible induction of anomalous splicing based on the single variations composing the haplotype.

**Results**

Data analysis highlighted statistically significant differences in frequencies of selected haplotypes (frequency >5% in at least one population) between populations with different clinical manifestations. Our study shows that some haplotypes are more frequent in target populations compared to controls; in particular, one haplotype marks 63% of FC-PI, 33% of FC-PS, 36% of CFTR-RD, 20% of CBAVD, 13% of ISHV, 3% of GC and 6% of NC alleles. No anomalous splicing was produced by the individual sequence variations. Studies concerning the modulation of the CFTR mRNA levels by the presence of selected haplotypes in populations under investigation are still running.

**Conclusions**

Our results confirm that the simultaneous presence in *cis* of multiple variations composing particular haplotypes is characteristic of some CF and CF-like populations in respect to controls. We hypothesize that the presence of a specific haplotype, rather than single variations, can affect the processing of the primary transcript of the CFTR, reducing its level. Moreover, haplotypes may be markers of mutated alleles that may be associated with different clinical manifestations. The use of haplotypes can be the basis of broad-spectrum screening programs, with a considerable saving of costs and reduction of investigation times.

## P4 Cystic fibrosis with residual function mutations in italy: epidemiology and clinical characteristics

### Donatello Salvatore^1^, Rita Padoan^2,4^, Roberto Buzzetti^3^, Annalisa Amato^4,5^, Barbara Giordani^4,5^, Gianluca Ferrari^4,6^, Fabio Majo^4,7^

#### ^1^Centro Regionale Fibrosi Cistica, AOR Ospedale San Carlo, Potenza, Italy; ^2^Centro regionale di supporto per la fibrosi cistica, ASST Spedali Civili, Brescia, Italy; ^3^Pediatra, epidemiologo, Bergamo, Italy; ^4^Italian Cystic Fibrosis Registry, Rome, Italy; ^5^Lega Italiana Fibrosi Cistica ONLUS, Rome, Italy; ^6^Centro Nazionale Malattie Rare, Istituto Superiore di Sanità, Rome, Italy; ^7^Centro Fibrosi Cistica, Ospedale Pediatrico Bambino Gesù, Rome, Italy

##### **Correspondence:** Donatello Salvatore (saverdon@gmail.com)

**Background**

The US-FDA expanded approved use of Ivacaftor to 28 mutations determining a residual functioning (RF) CFTR protein. This decision let about 1,500 subjects with CF (about 5% of US patients) to treat with Ivacaftor. The European Registry 2014 report described the rates of a few RF variants (3849+10kbC→T in 0.82% of tested alleles, 2789+5G→A in 0.95%, D1152H in 0.52%). Italian Registry reported about 10% of patients carrying one of these mutations in 2014. The high prevalence of these mutations and the particular Italian genetic background encourage to describe prevalence and phenotype of RF variants.

**Materials and Methods**

A query was made to the Italian Registry about the patients carrying the mutations: 2789+5G→A, 3849+10kbC→T, 3272-26A→G, 711+3A→G, E56K, P67L, R74W, D110E, D110H, R117C, L206W, R347H, R352Q, A455E, D579G, E831X, S945L, S977F, F1052V, R1070W, F1074L, D1152H, D1270N, K1060T, E193K, A1067T, G1069R, R1070Q. Data (2015 database) were retrieved about diagnosis, nutrition, lung function, complications, microbiology. Results of RF patients were compared with those of F508del homozygous subjects.

**Results**

Main results are shown in Table 5. 780 subjects were identified over a total of 5204 (15%); males were 392 (50.3%), mean age was 27.3 years. Diagnosis was made meanly at 13.7 years, with a mean sweat chloride of 75.8 mmol/l, by symptoms (55.8%), screening (26.8%), familiarity (11.3%) and male infertility (4.7%); meconium ileus was rare (0.8%). Nutrition is good with mean BMI z-score 0.3 in children and mean BMI 23.3 kg/m^2^ in adults. Pneumopathy is characterized by mean FEV_1_ 83.7% predicted. Prevalence of chronic *Pseudomonas Aeruginosa* was 26.9%, whereas *Staphylococcus Aureus* was present in 45.4% of patients. The most frequent complication was liver disease (11.9%), whereas diabetes was rare (2.9%). These data are similar in subjects whose genotype shows the presence of the RF mutation in heterozygosis with F508del or with another different mutation. Subjects with more frequent (N > 30) genotypes (double heterozygosis of F508del with 3849+10kbC→T, 2789+5G→A and D1152H, respectively) showed similar clinical features, but lung disease is more severe in subjects F508del/3849+10kbC→T. F508del homozygous patients showed younger age at diagnosis, meconium ileus in 12.2% of cases, and worse results as regards nutrition, lung disease and prevalence of complications.

**Conclusions**

Patients with RF of CFTR protein are numerous in Italy and have a milder phenotype than F508del homozygous. Lung disease is present, although it can be delayed in onset in most of these patients, but it may became severe in some.

**Table 5:** **(abstract P4).** Main results of subjects with RF mutations and F508del homozygous

**Year 2015**									
**Patients with at least one RF mutation**					**Controls: F508del homozygous**				
**Parameters**	**N**				**Parameters**	**N**			
**Total**	780	%			**Total**	1.096	%		
**Gender**					**Gender**				
M	392	50,3			M	591	53,9		
F	388	49,7			F	505	46,1		
		**Mean**	**Median**	**Range**			**Mean**	**Median**	**Range**
**Age at 2015 Dec, 31**	780	27,3	27,2	0,1-5,5	**Age at 2015 Dec, 31**	1.096	20,8	19,8	0,3-8,4
**Age at diagnosis**	767	13,7	6,7	0 - 65,2	**Age at diagnosis**	1.086	1,9	0,2	0-37,9
**Sweat chloride (mmol/L)**	719	75,8	76	12-170	**Sweat chloride (mmol/L)**	936	101,4	100	7,5-70
**Diagnosis by**		%			**Diagnosis by**		%		
Symptoms	431	55,8			Symptoms	523	48,6		
Screening	207	26,8			Screening	379	35,2		
Familiarity	87	11,3			Familiarity	30	2,8		
Male infertility	36	4,7			Male infertility	1	0,1		
Meconium Ileus	6	0,8			Meconium Ileus	134	12,4		
Prenatal Diagnosis	6	0,8			Prenatal Diagnosis	10	0,9		
**Nutrition**					**Nutrition**				
BMI z_score 2-18 years	247	0,3	0,4	-3,4-2,5	BMI z_score 2-18 years	444	-0,4	-0,3	-4,4-3,4
BMI ≥18 years	466	23,3	22,9	15,1-39,1	BMI≥ 18 years	580	21,1	20,9	13,4-41,3
**Lung function**					**Lung function**				
FEV1 % predicted	594	83,7	89,2	16,1 - 141,9	FEV1 % predicted	789	74,3	76,5	14-135,6
FVC % predicted	577	91,4	92,8	34,2 - 175,6	FVC % predicted	738	87,1	89,5	13,7-148,9
**Microbiology**		**%**			**Microbiology**		**%**		
Pseudomonas Aeruginosa	198	26,9			Pseudomonas Aeruginosa	448	43,3		
Staphylococcus Aureus	334	45,4			Staphylococcus Aureus	588	56,9		
Non Tuberculus Mycobacteria	4	0,5			Non Tuberculus Mycobacteria	8	0,8		
**Complications**		%			**Complications**		%		
ABPA	6	0,9			ABPA	41	4,4		
Diabetes	22	2,9			Diabetes	260	23,9		
Pneumothorax	0	0			Pneumothorax	6	0,6		
Liver disease	91	11,9			Liver disease	317	29,1		
Haemoptysis	7	0,9			Haemoptysis	22	2,0		
Neoplasms	2	0,3			Neoplasms	5	0,5		

## P5 A new poly-T allele of the *CFTR* gene

### Cecilia Surace^1^, Marco Lucarelli^2,3^, Valentina M Sofia^1^, Fabio Majo^4^, Giuseppe Cimino^5^, Silvia Pierandrei^2,6^, Nicola Ullmann^7^, Vincenzina Lucidi^3^, Antonio Novelli^1^, Adriano Angioni^1^.

#### ^1^UOC Laboratorio Genetica Medica, Ospedale Pediatrico “Bambino Gesù”, Rome, Italy; ^2^Dip. di Biotecnologie Cellulari ed Ematologia, Sapienza Università di Roma, Rome, Italy; ^3^Istituto Pasteur Fondazione Cenci Bolognetti, Sapienza Università di Roma, Rome, Italy; ^4^UOC Fibrosi Cistica, Ospedale Pediatrico “Bambino Gesù”, Rome, Italy; ^5^Centro di Riferimento Regionale Fibrosi Cistica, Azienda Policlinico Umberto I, Rome, Italy; ^6^Centro di Riferimento Regionale Fibrosi Cistica, Azienda Policlinico Umberto I, Rome, Italy; ^7^UO Broncopneumologia, Ospedale Pediatrico “Bambino Gesù”, Rome, Italy

##### **Correspondence:** Cecilia Surace (cecilia.surace@opbg.net)

**Background**

The poly-T tract, located at the junction of intron 8 (IVS-8) and exon 9, is well known. It influences transcription, and thereby reduces the amount of normal CFTR protein. The number of T residues present, 5, 7 or 9, affects the splicing efficiency of exon 9. If the T5 allele is present, a proportion of CFTR transcripts will lack exon 9, which produces a nonfunctional protein and variable CF symptoms. The TG repeats, 5’ of the poly-T, also influence splicing of exon 9, and when present on the same allele as a 5T repeat, the longer the TG-repeats, the higher the proportion of CFTR transcripts that will lack exon 9. In this report we describe the finding of a new (TG)13T6 allele.

**Materials and methods**

In this study, we described a pregnant 36 years old woman, who has come to our attention for CF screening. The analysis has been performed by means of Next Generation Sequencing with Multiplicom MASTR™ Dx (Multiplex Amplification of Specific Targets for Resequencing) using MiSeq platform. Data analysis has been carried out by Sophia Data Driven Medicine (Sophia DDM), Sophia Genetics®. In addition, we performed a nasal brushing of the patient. RNA was extracted, reverse transcribed and amplified using standard methods. RT-PCR results were analyzed by a semiquantitative densitometric assay.

**Results**

The genomic analysis did not reveal any known pathogenic variant, but it showed an atypical poly-T/TG repeats genotype. The patient was characterized as c.[1210-12T[6];1210-34TG[13]];[1210-12T[7];1210-34TG[12]]. It was not possible to disclose if the allele (TG)13T6 was arisen *de novo* because the patient’s mother was not informative and the father was not available. To better understand the functional consequences of the genotype (TG)13T6/(TG)12T7, it was compared with the homozygous genotype (TG)12T7/(TG)12T7. The densitometric values of the homozygous genotype were used to calculate the contribution of the (TG)13T6 allele within the (TG)13T6/(TG)12T7 genotype. The amount of wild-type mRNA from (TG)13T6 allele resulted to be slightly higher (29.9%) than exon 9- abnormally spliced CFTR mRNA (20.1%).

**Conclusions**

Data obtained from RNA study suggest that the residual quantity of functional CFTR mRNA is high enough to allow to consider the genotype (TG)13T6/(TG)12T7 (and the allele (TG)13T6) non pathogenetic for CF and CFTR related disorders (CFTR-RD).

**Consent for publication:** The patient gave the consent to publish the clinical data.

## P6 Qualitative and quantitative evaluation of alternative splicing products using the digital droplet PCR

### Marika Comegna^1,2^, Antonella M Di Lullo^2,4^, Renato Liguori^1,2^, Francesca Manzoni^1,2^, Chiara Di Palma^1,2^, Sabrina Maietta^1,2^, Federica Zarrilli^3^, Felice Amato^1,2^

#### ^1^Department of Molecular Medicine and Biotechnology, University of Naples Federico II, Naples, Italy; ^2^CEINGE- Advanced Biotechnology, Naples, Italy; ^3^Department of Biosciences and Territory, University of Molise, Isernia, Italy; ^4^Department of Neuroscience, ENT Section, University of Naples Federico II, Naples, Italy

##### **Correspondence:** Felice Amato (felice.amato@unina.it)

**Background**

The CFTR gene encodes for a chlorine-transporting protein essential for the correct hydration of epithelium in many organs and tissues. The malfunction of CFTR protein causes Cystic Fibrosis, a genetic disease with an incidence of about 1 out of 3000 births. To date, more than 2000 CFTR gene mutations are known and many of them alter the correct splicing of its mRNA. The qualitative evaluation of the effect of a splicing genetic variant is a simple procedure that by an RT-PCR followed by an electrophoretic separation can reveal the alternative splicing products. Conversely, making a quantitative and accurate evaluation of splicing products, in order to evaluate the percentage of proper residual splicing, requires complicated and expensive procedures.

**Materials and methods**

We evaluated the use of digital droplet PCR (ddPCR) with EVAGreen technology to quantify the amount of exon skipping due to splicing mutations. In particular, we used total RNA extracted from nasal cells brushed directly from subjects bearing different intron 9 polyT variants, including the 5T-12TG allele. The total RNA was retro-transcribed and amplified by ddPCR using a single primer pair to amplify both PCR products, 305 bp (correct splicing) and 122 bp (Exon 10 skipped).

**Results**

Using a single primer pair it was possible to determine different percentage of exon 10 skipping depending on the poly T tract size, as reported in literature. In particular, we obtained about 50% and 95% correct splicing from 5T/7T and 7T/7T subjects.

**Conclusions**

These data, even if preliminary, strongly suggest that it is possible to accurately quantify the percentage of correct residual splicing with a simple pair of primers, without the use of special probes. This methodology could be helpful especially in the analysis of composite heterozygous, in which splicing mutations are associated with other severe mutations and it is necessary to know the exact contribution of the splicing variant to the phenotype.

**Acknowledgements**

We acknowledge Ministero della Salute (Rome, Italy) L.548/93 for the regional research funding quote of years 2007-12.

## P7 Mutations in genes involved in the pancreatic pathway are a risk factor for pancreatitis in Cystic Fibrosis patients

### Valentina M Sofia^1^, Cecilia Surace^1^, Vito Terlizzi^2^, Federico Alghisi^3^, Antonella Angiolillo^4^, Cesare Braggion^5^, Natalia Cirilli^6^, Carla Colombo^7^, Antonella M Di Lullo^8,9,10^, Rita Padoan^11^, Serena Quattrucci^12^, Valeria Raia^13^, Giuseppe Tuccio^14^, Federica Zarrilli^15^, Antonio Novelli^1^, Vincenzina Lucidi^3^, Marco Lucarelli^16,17^, Giuseppe Castaldo^8,9^, Adriano Angioni^1^

#### ^1^UOC Laboratorio Genetica Medica, Ospedale Pediatrico “Bambino Gesù”, Rome, Italy; ^2^Dipartimento di Pediatria, Centro Regionale Toscano per la Fibrosi Cistica, Azienda Ospedaliero-Universitaria Meyer, Florence, Italy; ^3^UOC Fibrosi Cistica, Ospedale Pediatrico “Bambino Gesù”, Rome, Italy; ^4^Centre for Research and Training in Medicine for Aging, Department of Medicine and Health Sciences "Vincenzo Tiberio", University of Molise, Campobasso, Italy; ^5^Cystic Fibrosis Center, Anna Meyer Children's Hospital, Florence, Italy; ^6^Dipartimento Materno-Infantile, Ospedali Riuniti Ancona, Centro Regionale Fibrosi Cistica, Ancona, Italy; ^7^Centro Regionale Fibrosi Cistica, Fondazione IRCCS Ca' Granda, Ospedale Maggiore Policlinico, Università degli Studi di Milano, Milan, Italy; ^8^CEINGE-Biotecnologie Avanzate, Naples, Italy; ^9^Dipartimento di Medicina Molecolare e Biotecnologie Mediche, Università di Napoli Federico II, Naples, Italy; ^10^Dipartimento di Neuroscienze, Sezione di ORL, Università di Napoli Federico II, Naples, Italy; ^11^Cystic Fibrosis Support Center, Paediatric Department, Children's Hospital, AO Spedali Civili, Brescia, Italy; ^12^Centro Fibrosi Cistica, Sapienza Università e Policlinico Umberto I, Rome, Italy; ^13^Centro Regionale Fibrosi Cistica, Sezione Pediatrica, Dipartimento di Scienze Mediche Traslazionali, Università di Napoli Federico II, Naples, Italy; ^14^Centro di Riferimento per la Fibrosi Cistica - Regione Calabria, Soverato, Italy; ^15^Dipartimento di Bioscienze e Territorio, Università del Molise, Isernia, Italy; ^16^Dip. di Biotecnologie Cellulari ed Ematologia, Sapienza Università di Roma, Rome, Italy; ^17^Istituto Pasteur Fondazione Cenci Bolognetti, Sapienza Università di Roma, Rome, Italy

##### **Correspondence:** Valentina M Sofia (valentinamaria.sofia@opbg.net)

**Background**

Mutations of *CFTR* gene, responsible of Cystic Fibrosis (CF), cause abnormal chloride transport in the airways, pancreas, intestine, and vas deferens, leading to progressive lung and pancreatic dysfunction, elevated sweat electrolyte levels and male infertility, respectively. Chronic pancreatitis (CP) is a complication of CF due to prematurely activated trypsin within the pancreas that plays a pivotal role in triggering the activation cascade of pancreatic digestive zymogens. We studied several genes belonging to different pancreatic pathways to evaluate if mutations in such genes may represent risk factors for CP also in CF patients.

**Materials and methods**

We enrolled: 48 patients affected by CF and pancreatitis, recruited through a multicentric study; 35 patients with CF without symptoms nor history of pancreatitis; 80 unrelated healthy controls.

We designed a panel of 8 genes involved in the intrapancreatic activation of trypsin *PRSS1, PRSS2, SPINK1, CTRC, CASR, CFTR, CTSB* and *KRT8* and 24 additional genes classified into four groups according to the activity of the encoded protein in the “Pancreatic Secretion Pathway” (PSP).

Targeted resequencing was performed using a TruSeq Custom Amplicon Low Input technology (Illumina) with the MiSeq platform. All identified variants were analyzed with bioinformatic softwares evaluating the impact of the change in amino-acidic structure on protein functionality. The variants predicted as “damaging” by at least three of these softwares were validated by standard Sanger sequencing. The study obtained the approval of local ethical committees.

**Results**

We found 14 patients (29.16%) with mutations in genes involved in the intra-pancreatic activation of trypsin in the group of CF patients with CP, while mutations in such genes were found in 2/35 (5.7%) patients with CF without experience of CP and in 3/80 (3.8%) healthy subjects (chi square, *p*<0.001). Thus, we found mutations in 12 genes of 11 patients for the PSP in the cohort of patients with CF and CP. Finally, 14/48 patients (29.2%) showed one or more mutations in the genes involved in the intra-pancreatic activation of trypsin and 6 patients had also mutations in the genes involved in PSP. Of the remaining 34 patients, 5 presented mutations in at least one gene of the other pancreatic pathways of the panel.

**Conclusions**

This study suggests that the trans-heterozygous association between *CFTR* mutations and the genes involved in the pathway of pancreatic enzyme activation may be a risk factor and predispose to the development of pancreatitis in patients with CF.

## P8 Nutritional disorders in a cohort of pediatric CF patients: experience and considerations from Brescia Cystic Fibrosis Centre

### Valentina Tradati, Eliana di Stefano, Rita Padoan

#### Cystic Fibrosis Support Centre, Department of Paediatric Children’s Hospital, ASST- Spedali Civili, Brescia, Italy

##### **Correspondence:** Valentina Tradati (valentina.tradati@gmail.com)

**Background**

Transient nutritional disorders are common pathological conditions during childhood, especially when children progress throughout critical steps of their development. About 25% of children with a normal physical and mental development can indeed be affected by nutritional disorders. This percentage increases to 35% in children with developmental defects. Temporal continuity and the tendency to persist might determine the evolution of the difficulties in a real food disorder [1,2]. Cystic fibrosis (CF) diagnosis can be a significant risk factor in the development of the primary relationship between caregivers and children, favouring anxiety and eating difficulties.

**Materials and Methods**

A qualitative research on nutritional issues and behavioural during meals was performed by the psychologist and the dietitian in CF pancreatic insufficient patients (aged 0 to 18 years). Clinical charts were reviewed and BMI centiles were recorded. In 0-2 years group weight/length centiles were collected. The study obtained the approval of local ethical committee.

**Results**

Seventy seven patients were selected for this study, their ages being: 0-2 years old: 8 patients; 3-5 years: 14 patients; 6-10 years old: 21 patients; 11-18 years old: 34 patients. 12% of parents reported inadequate behaviours during the main meals or described the necessity of using non canonical methods to feed their children (i.e., distraction, forced feeding); moreover 38% of parents described an anxious status correlated to their children’ weight gain and referred to the moment of the weight check during follow up clinical visits.In Table 6 nutritional status and frequencies of eating disorders are showed in the different age groups.

**Conclusions**

The data analysed in our Centre showed that nutritional disorders are common pathological conditions among CF patients and they need to be considered carefully. Symptoms detected during the early childhood do not physiologically resolved and they have the tendency to persist during puberty and adolescence. Eating difficulty does not concern only patients with pathological BMI or at risk of malnutrition; we observed children with a normal BMI who are affected by mild or serious nutritional disorders and parents seriously worried about their child's growth, although the presence of a normal BMI. The high incidence of nutritional disorders among paediatric patients affected by cystic fibrosis highlights the importance of this topic. The medical staff with the help of the psychologist and the dietitian should carefully discuss possible strategies to avoid the onset of these conditions since the time of first communication with parents after CF diagnosis and during follow up.

**References**

1. Trombini E. Il cibo rifiutato. I disturbi alimentari precoci e la Giocoterapia focale con i bambini e I genitori. Pendragon, Bologna 2010.
2. Chatoor I. Feeding and other disorders of Infancy or Early Childhood, in Tasman A, Kay J, Lieberman L. Psychiatry, Saunders, Philadelphia 1996.

**Table 6:** **(abstract P8).** See text for description

Cbmi				Eating Disorders			
Age ranges (years)	<3	3-10	10-25	Lack of appetite	Food Tightness	Vomiting	Difficulties in chewing
0-2*	0%	12,5%	25%	12,5%	0%	12,5%	12,5%
3-5	14,3%	0%	14,3%	42,9%	35,7%	7,1%	21,4%
6-10	19 %	14,3%	14,3%	33,3%	9,5%	4,8%	0%
11-18	5,9%	14,7%	20,6%	17,6%	11,8%	0%	0%

*in this age group weight/length centiles were calculated

## P9 DHA and Cystic Fibrosis: six months of treatment in a cohort of cystic fibrosis patients

### Mirella Collura^1^, Patrizia Dato^1^, Maria G Sciarrabone^2^, Carmela Fondacaro^2^, Lisa Termini^1^, Annalisa Ferlisi^1^, Maria A Orlando^1^, Gabriella Traverso^1^, Marcella Bertolino^1^, Francesca Ficili^1^

#### ^1^U.O. Pediatria II per la FIBROSI CISTICA (CRR) e le Malattie Respiratorie. ISMEP, Palermo, Italy; ^2^Dipartimento di Scienze per la promozione della salute materno infantile G. D’Alessandro. Università degli studi di Palermo, Palermo, Italy

##### **Correspondence:** Francesca Ficili (fficili@hotmail.com)

**Background**

Nutrition is a main feature of the management of patients affected by Cystic Fibrosis (CF) and their nutritional status is directly related to pulmonary function and survival. Several studies suggest that a diet implemented with omega 3 fatty acid, such as Docosahexaenoic Acid (DHA), shows some anti-inflammatory effects, attenuates pulmonary and gastrointestinal symptoms, reduces the disease progression.

**Materials and methods**

DHA basic has been introduced as a nutritional supplement into the diet of 26 CF patients (12 males and 14 females) with an average age of 11,5 years affected by loss of weight and poor growth. All patients have been followed-up for 6 months monitoring weight, BMI, FEV1 and pulmonary exacerbations.

**Results**

We found an average weight gain of 3,5 kg in 22 patients, whereas 4 patients did not show any weight modification. Spirometry has been performed on 12 patients, 6 of whom had an improved FEV1 with an average increase of 7%, 6 did not presented any change. Only 6 cases of pulmonary exacerbations, requiring hospitalization, have been observed.

**Conclusions**

Diet integration with DHA basic can be certainly considered as a part of the management of CF patients, because of the improvement of their nutritional and respiratory status. Our study strenghtens the evidences yet published about the anti-inflammatory effects of DHA, demonstrating both an improvement of auxological parameters and pulmonary symptoms, and a decrease of exacerbation episodes and subsequently of the use of antibiotics therapy.

**Consent for publication:** The patients gave the consent to publish clinical data.

## P10 Moraxella catarrhalis in Cystic Fibrosis children: a retrospective study

### Federico Cresta^1^, Valentina Baglioni^1^, Silvia Garuti^2^, Isabella Buffoni^1^, Francesca Landi^1^, Rosaria Casciaro^1^, Laura Minicucci^1^

#### ^1^Cystic Fibrosis Center, Pneumology Unit, IRCCS G. Gaslini Institute, Genova, Italy; ^2^Pneumology Unit, Policlinico San Martino - IST, Genova, Italy

##### **Correspondence:** Silvia Garuti (silvia_garuti@llibero.it)

**Background**

*Moraxella catarrhalis* (*Mc*) is a human-restricted opportunistic bacterial pathogen of the upper respiratory mucosa. It frequently colonizes the nasopharynx asymptomatically. The role of this microorganism in Cystic Fibrosis (CF) patients is not well established. The aim of this retrospective study is to define the incidence of *Mc* colonization in CF children population and to evaluate the role of antibiotic therapy in *Mc* eradication.

**Materials and methods**

In our study we enrolled 54 CF children (23 females and 31 males; 0-12 years old, mean age 6.4 years). Periodic nasal and pharyngeal swabs were performed in every enrolled patient for 2 consecutive years every 3 months, during routine outpatient visits. For every *Mc+* patient, we collected data regarding presence of nasal symptoms (rynitis, polyposis, adenoidal hypertrophy), development of relapse and relapse timing. In 19 of the 35 *Mc+* patients, we performed eradication antibiotic therapy with oral amoxicillin/clavulanate course for 15 days.

**Results**

*Mc* was isolated in 35 children 64.8% (17 females and 18 males, mean age 6.1 years), 24/35 (68.6%) in nasal swab, 9/35 (25.7%) in pharyngeal swab and in 2/35 (5.7%) in both swabs simultaneously. From the collected data we can deduce that:

1) 29/35 patients (82.9%) relapsed;

2) in 16/19 patients treated (84,2%) we observed a relapse *versus* 13 relapses observed in non-treated group (16 patients, 81%);

3) we did not found a significant statistical correlation between factors analyzed (sex, swab type, *Mc* beta lactamase resistance, eradication attempt, ORL symptoms) and relapse;

4) relapses was more frequent in females (88.2% female vs. 77.8% male) and in subjects with nasal *Mc+* and nasal symptoms;

5) the median relapsing time was 9 month in eradicated patients and 22 months in non-treated group.

**Conclusions**

Our study can allow us to postulate that:

1. *Mc* is isolated more frequently in nasal swab than in pharyngeal swabs;
2. Nasal *Mc+* and nasal symptoms resulted to be associated with a more frequent and earlier relapse;
3. Oral amoxicillin/clavulanate eradication therapy for *Mc* is not useful to prevent microbiological and clinical relapse in CF children.

Further study on larger populations will be crucial in order to better define *Mc* incidence and its ideal management in CF children.

**Consent for publication:** The patients gave the consent to publish clinical data.

## P11 Burkholderia Cepacia Complex Infection in patients with Cystis Fibrosis: molecular typing and therapeutic strategies

### Daniela Girelli^1^, Antonio Teri^1^, Samantha Sottotetti^1^, Arianna Biffi^1^, Chiara Vignati^1^, Monica D’accico^1^, Anna Maraschini^1^, Milena Arghittu^1^, Carla Colombo^2^, Giovanna Pizzamiglio^3^, Elisa Cariani^1^

#### ^1^UOS Microbiology and Cystic Fibrosis Microbiology, Fondazione IRCCS Ca' Granda Ospedale Maggiore Policlinico, Milano, Italy; ^2^UOC Fibrosi Cistica Pediatrica, Cystic Fibrosis Centre, Fondazione IRCCS Ca' Granda Ospedale Maggiore Policlinico, Milano, Italy; ^3^ Respiratory Disease Department, Cystic Fibrosis Center Adult Section, Fondazione IRCCS Ca' Granda Ospedale Maggiore Policlinico Milano, Milano, Italy

##### **Correspondence:** Samantha Sottotetti (Samantha.sottotetti@hotmail.it)

**Background**

*Burkholderia cepacia* complex (Bcc) is an opportunistic microrganism causing severe respiratory infection in Cystic Fibrosis (CF) patients. To Bcc belong 17 Genomovars, each with different pathogenic properties. Several resistance mechanisms make eradication of Bcc almost impossible and it is difficult to establish a correct therapeutic scheme since, to date, there is no method considered "Gold Standard" for in vitro determination of antibiotics sensitivity, nor MIC breakpoints specific for Bcc. Aims: conduct a retrospective survey to define prevalence of Bcc among patients attending the CF Center in Milan; assess frequency of the most isolated Genomovars and its variation throughout time; compare three different methods to determine the minimum inhibitory concentration (MIC) of several antibiotics.

**Materials and methods**

We focused on 24 patients (mean age 29 years) chronically colonized with Bcc, out of the 838 attending our centre by the end of 2015. Biochemical tests and a subsequent recA gene sequencing were carried out to identify Genomovars of 151 strains of Bcc isolated from the low respiratory tract of these patients over the period between 2005 and 2015 (one strain per year per patient since first colonization). Antibiotic sensitivity tests were performed on 51 of these strains for six antibiotics with two manual methods, microdilution scalar redoubling (MSR) and Epsilometric E-Test, and an automated Microdilution Method (MicroScan WalkAway plus System, Beckman Coulter).

**Results**

Bcc prevalence value has increased steadily over the study period from 1.1% in 2005 to 2.9% in 2015, and 7 out of the 17 genomovars known today were recognized. The prevalence values of the different subspecies are: 45.8% for *B.cenocepacia* Genomovar III, 16.7% for *B.stabilis* Genomovar IV, 12.7% for *B.multivorans* Genomovar II, 8.34% for *B.cepacia* Genomovar I and *B.vietnamensis* Genomovar V, 4.16% for *B.seminalis* BCC7 and *B.metallica* BCC8. All patients maintained the same genomovar acquired at first infection. No significant correlation for any antibiotic was found among MIC values obtained through the three different chemosensitivity methods.

**Conclusions**

Bcc prevalence values observed over the years are in line with literature data. In Milan CF centre, infections are mainly sustained by *B.cenocepacia* Genomovar III, and all patients maintained the same Genomovar acquired at first infection, as the retrospective analysis shows. We assess the importance of a timely molecular typing of Bcc strains to guide the clinician dealing with CF colonized patients. The chemosensitivity results are consistent with EUCAST remarks in regard to poor reproducibility and therefore reliability of the tests.

## P12 Importance of upper airways in early *P. Aeruginosa* infection in CF

### Daniela Dolce^1^, Novella Ravenni^1^, Silvia Campana^1^, Erica Camera^1^, Carlo Castellani^2^, Riccardo Guarise^2^, Cesare Braggion^1^, Giovanni Taccetti^1^

#### ^1^Cystic Fibrosis Centre, Department of Pediatric Medicine, Meyer Children Hospital, Florence, Italy; ^2^Rehabilitation Unit, Meyer Children Hospital, Florence, Italy

##### **Correspondence:** Daniela Dolce (d.dolce@meyer.it)

**Background**

Chronic *Pseudomonas aeruginosa* (PA) lung infections are the major cause of morbidity and mortality in cystic fibrosis (CF) patients. During the initial phase of infection, patients can be treated with early eradication therapy to prevent or delay chronic PA lung infections. Following eradication, lung re-infection can occur with bacteria with the identical genotype. This may be due to re-colonization from the patient's paranasal sinuses. Although the CFTR defect equally affects respiratory cells in the upper airways (UAW) and lower airways (LAW), the microbiological assessment of UAW is not included in the standard of care. The aims of the study were to evaluate, using non-invasive methods, the microbiological status of UAW of CF patients not chronically infected with PA.

**Materials and methods**

During the period 2014-2017, 47 patients not chronically infected by PA, according to the Leeds’ definition, were evaluated. We simultaneously sampled the LAW by expectorated sputum or deep throat swab and the UAW by nasal lavage, using the Mainz method. Genotyping of PA isolated from UAW and LAW were also performed by BOX-PCR in order to assess if the UAW may represent a re-infection source.

**Results**

A total of 64 nasal lavages and concomitant LAW specimens were analyzed from 47 patients (median age 13 years, range 2-48). During the study period 14 out of 64 (22%) nasal lavage and 32 (50%) LAW were found positive for PA. Positive samples were found simultaneously from upper and lower airways in 10 (15.6%) out 64 specimens and they carried identical PA genotypes in the two compartments. In 4 patients PA was cultured from the UAW after successful eradication therapy.

PA isolates from UAW showed high susceptibility to the majority of tested antibiotics. No resistance to colistin, meropenem or ceftazidime was found. Only 2 PA isolate showed a mucoid phenotype.

**Conclusions**

The presence of PA isolates in the nasal lavage suggests that the UAW can play a role in the acquisition and/or persistence of these bacteria, even after eradication therapy, this may be due to the fact that the UAW is undertreated in CF. Since the PA strains isolated from the UAW had low antibiotic resistance, the efficacy of antibiotic treatment of the paranasal sinuses should be evaluated. In summary, upper airway involvement requires prospective investigation and an interdisciplinary consensus on diagnosis and therapy.

Supported by FFC# 30/2015

## P13 Prevotella species in the lower airways of Cystic Fibrosis patients

### Patrizia Morelli, Eleonora Calderone, Roberto Bandettini

#### Microbiology Laboratory of CF - Giannina Gaslini Institute – Genoa, Italy

##### **Correspondence:** Patrizia Morelli (patriziamorelli80@libero.it)

**Background**

Recent microbiome studies suggest that the airways of individuals with cystic fibrosis (CF) are colonized by a complex microbiota, including strict anaerobes that traditionally inhabit the oral cavity. *Prevotella* species are consistently one of the most prevalent members of the CF microbiome, however few studies with cultural methods have investigated the antibiotic resistance of these specie in CF. Exposure to antibiotics used routinely in the treatment of CF pulmonary infection may also increase the resistance to antimicrobials even in anaerobic strains. Therefore in this study, we determined the abundance and antimicrobial susceptibility of *Prevotella* isolates in a cohort of CF patients at Giannina Gaslini Institute.

**Materials and methods**

Anaerobic and aerobic culture techniques were used to culture sputum samples from 60 (43 adult and 17 pediatric) CF patients. Sputum (20μl) were plated onto Brucella Blood Agar with Hemin and Vitamin K1 (BD) and Schaedler Kanamycin-Vancomycin Agar with 5% Sheep Blood (BD). The plates were incubated under anaerobic conditions at 37 °C for 5-7 days then were examined and unique isolates were enumerated and subcultured. The specie identification were performed in duplicate by matrix-assisted laser desorption ionization-time of flight (MALDI-TOF) using a Vitek MS Spectrometer (bioMérieux). To assess the susceptibility of the obligate anaerobe isolates to antibiotics, was used the Thermo Scientific™Sensititre™ Anaerobe MIC Plate. Interpretative criteria for susceptibility to antibiotics were in accordance with Eucast.

**Results**

We found 70 strains of *Prevotella*: 39 *P. Melaninogenica* (56%), 10 *P. Denticola* (14%), 7 *P. Nigrescens* (10%), 7 *P. Salivae* (10%), 3 *P. Intermedia* (4,3%), 2 *P. Oralis* (2,9%), 1 *P. Baronie* (1,4%) and 1 *P. Disiens* (1,4%). The strains analysed show resistance to Penicillin (MIC90 >8 mg/L), amoxicillin (MIC90 > 32 mg/L), clindamycin (MIC90 > 64 mg/L) and piperacillin (MIC90 = 64 mg/L). All strains were susceptible to metronidazole (MIC90 = 4 mg/L), imipenem (MIC90 = 0.12 mg/L) and piperacillin/tazobactam (MIC90 ≤ 16 mg/L).

**Conclusions**

Our study confirms an abundance of *Prevotella* in lower airway of CF patients. Regarding the antimicrobial susceptibility, previous studies on isolates from CF patients described a high resistance to amoxicillin and macrolides. Our results demonstrate that metronidazole, meropenem/imipenem and piperacillin/tazobactam are likely to be most effective against *Prevotella*. Further research into anaerobic pathogens, in particular *Prevotella,* will lead to improve treatments to reduce the severity of CF lung disease.

## P14 The challenge of macroscopic sputum quality assessments with a colour grading system in Cystic Fibrosis patients

### Riccardo Guarise^1^, Chiara Degli Innocenti^1^, Chiara Castellani^1^, Eleonora Masi^1^, Vito Terlizzi^2^, Maria Chiara Cavicchi^2^, Beatrice Ferrari^1^

#### ^1^Rehabilitation Unit, Meyer Children Hospital, Florence, Italy; ^2^Cystic Fibrosis Centre, Department of Pediatric Medicine, Meyer Children Hospital, Florence, Italy

##### **Correspondence:** Riccardo Guarise (riccardo.guarise@meyer.it)

**Background**

Lung disease in CF is characterized by airway inflammation, persistent cough with production and stagnation of thick mucus. Sputum samples are a standard part of the clinical assessment of CF patients and are routinely collected for microbial surveillance and respiratory physiotherapy evaluation. Macroscopic sputum assessment, which consists primarily on visual examination of volume, colour, apparent viscosity and patient-perceived thickness, is a subjective measurement with a potentially high variability. The aim of the study is to explore the feasibility and agreement of a 5-point colour scale for macroscopic evaluation of sputum colour.

**Materials and methods**

Clinically stable subjects with CF were recruited during their scheduled follow-up or the day before discharge from hospitalization. After performing a medical examination and spirometry patients were asked to withhold their usual bronchodilator therapy and start their usual airway clearance technique during which a sputum sample was collected by a respiratory physiotherapist (RPT) in a wide mouthed capped plastic container. The sample was assessed with a 5-point colour scale (from 0=lighter to 5=darker) by the patient, two RPTs and two residency doctors (RDs), which were blind to the patient identity, within ten minutes. Samples containing more than minimal salivary contamination were not evaluated. Sign test was used to test for consistent differences between patients and health professionals and within RPTs and RDs. Statistical significance was accepted at *p*<0.05. Sample characteristics are presented as mean (Table 7) unless specified.

**Results**

Nineteen subjects were recruited and a single sputum was collected per each. Six patients were excluded for excessive salivary contamination of the sample. Sputa from 13 subjects (7 females) aged 29(11) years with a FEV_1_ of 48,2(20,1%) pred. were assessed. Median colour point was the same within patients, RPTs and RDs (score=4). Sign test revealed agreement in colour assessment between patients and RPTs, patients and RDs, RPTs and RDs. Patients self-assessment scores were lower than the median of RPTs and RDs in 5(38%) subjects.

**Conclusions**

Although sputum properties and their changes are not correlated with frequency of infection, use of antibiotics, and quality of life, a sputum colour scale could be an objective useful tool to standardize the macroscopic description of sputum on medical record and progress notes among different health professionals. The study is still ongoing to evaluate agreement on a larger sample size along with a feasibility comparison with a 9-point colour scale.

**Table 7:** **(abstract P14).** Sample characteristics

Age, yrs	29(11)
Gender M/F	6/7
Body Mass Index (BMI), Kg/m^2^	20(3)
Pancreatic Insufficiency, n(%)	13(100%)
FEV1 % of predicted	48(20)
ΔF508 homozygosis, n(%)	5(38%)
Chronic infection by Pseudomonas Aeruginosa, n(%)	11(84%)

## P15 Molecular identification of persistent *Nocardia* infection by whole genome sequencing

### Ramona Pezzotta^1†^, Piercarlo Poli^2†^, Serena Messali^1^, Silvana Timpano^2^, Rita Padoan^2^, Erika Scaltriti^3^, Stefano Pongolini^3^, Simona Fiorentini^1^

#### ^1^Section of Microbiology, Dept. of Molecular and Translational Medicine University of Brescia/ASST - Spedali Civili, Brescia, Italy; ^2^Cystic Fibrosis Regional Support Centre, Children Hospital, ASST - Spedali Civili, Brescia, Italy; ^3^Istituto Zooprofilattico Sperimentale della Lombardia e dell’Emilia Romagna (IZSLER) “Bruno Ubertini” of Parma, Parma, Italy

##### **Correspondence:** Ramona Pezzotta (ramypezzotta@gmail.com)

^†^Equally contributed

**Background**

In the last few years, the range of lung infections due to emerging and atypical microorganisms has been widen by the increased availability of molecular diagnostic procedures. Pulmonary nocardiosis is a well-recognized cause of disease in patients with risk factors such as immunosuppression, malignancies, and severe lung disease. However, only sporadically, *Nocardia* spp have been considered pathogenic in CF. In this report we describe the identification by whole genome sequencing (WGS), of N. otitidiscaviarum as the cause of chronic pulmonary disease in BV, a 14 years-old CF girl.

**Materials and methods**

From October 2014, BV was admitted several times to the CF Centre in Brescia, for pulmonary exacerbations and, in May 2015, TC scan revealed the presence of pulmonary consolidations. To perform routine culture microbiological analyses, sputum and/or bronchoalveolar lavage (BAL) were sampled at each hospital admission. Samples were also subjected to direct gram staining and, in some cases were used for molecular procedures. Briefly, bacterial DNA was extracted using Qiagen kit and sequencing libraries were prepared with the Nextera XT sample preparation kit (Illumina, Inc., San Diego - California, USA). Sequencing was performed on the MiSeq and assembled with MIRA 4.0 using “accurate” settings for de-novo assembly mode.

**Results**

Despite the clinical condition, BV did not show infection by any canonical CF pathogens, whereas direct microscopy highlighted the presence of gram-positive filamentous bacteria compatible with *Actinomycetales*. Specific culture conditions (5 days on CAN agar in 5%CO_2_), applied on all the samples collected from May 2015 to October 2016, allowed to isolate bacteria and to perform antibiograms but not to identify the pathogen. WGS identified the isolates as belonging to the *Nocardia otitidiscaviarum* species and revealed that they were all clonal.

**Conclusions**

With no other pathogens detected, the repeated isolation of the same *Nocardia otitidiscaviarum* clone strongly suggest this bacterium as the sole responsible for the patient’s exacerbations. These data confirm that WGS is a useful tool for laboratory analysis during investigations on atypical CF infections sustained by fastidious organisms. It is important to underline that, in BV, specific treatment for Nocardia is efficient in obtaining symptoms remission; however, analogously to what occur in chronic *P. aeruginosa* infection, treatment does not eradicate the colonization. This suggests that both clinical and microbiological monitoring using WGS may be helpful to gain a deeper knowledge about the role of Nocardia infection in CF lung pathogenesis.

**Consent for publication:** The patients gave the consent to publish clinical data.

## P16 Non-tuberculous mycobacteria in adult patients with Cystic Fibrosis

### Silvia Bresci^1^, Lorenzo Corsi^2^, Riccardo Guarise^3^, Beatrice Borchi^1^, Annalisa Cavallo^1^, Filippo Bartalesi^1^, Massimo Pistolesi^2^, Alessandro Bartoloni^1^, Cesare Braggion^4^

#### ^1^Tropical and Infectious Disease Department, Careggi, Florence, Italy; ^2^Pneumology, Thoracic and Pulmonary Physiopathology Department, Careggi, Florence, Italy; ^3^Rehabilitation Unit, Meyer Children Hospital, Florence, Italy; ^4^Cystic Fibrosis Centre, Meyer Children Hospital, Florence, Italy

##### **Correspondence:** Silvia Bresci (silvia.bresci@gmail.com)

**Background**

Non-tuberculous mycobacteria (NTM) are ubiquitarious microorganisms with a borderline pathogenicity; NTM infection are often associated with an increased risk of morbidities in respiratory or systemic diseases and recent data suggests a potential increased frequency of NTM-positive cultures in adult patients with Cystic Fibrosis (CF). The aim of this study is to assess prevalence of NTM-positive culture and the incidence of NTM infections among our CF Centre casuistry.

**Materials and methods**

An observational retrospective analysis was carried out from 2010 to 2016 among adult CF patients. Respiratory function indexes (FEV_1_%pred, FVC, FEV_1_/FVC) and nutritional status (BMI) were collected within three months from first NTM isolation and un to one year from the start of treatment. The prevalences of the slow-growing Mycobacterium avium complex (MAC) and the rapid-growing Mycobacterium abscessus complex (MABSC) species were calculated. Simple isolations and non-tuberculous mycobacteria pulmonary disease (NTM-PD) were differentiated according to ATS/IDSA 2007 Statement. The efficacy of antimycobacterial therapy was assessed through radiographic imaging, changes in pulmonary function and sputum conversions at 3, 6 and 12 months from isolation (NTM) or from the onset of therapy (NTM-PD).

**Results**

One hundred fifty five CF adults were screened for NTM between 2010 to 2016. Among them, 23 patients (10 males), aged 30.5 (18-56) years with a FEV1 % pred. of 70.2 were found to have at least one positive sputum culture for NTM (prevalence of 15%); 41% of NTM positivity is due to MABSC and 38% to MAC. The overall prevalence for NTM-PD was 5% (8 patients) which were treated for NTM-PD with anti-mycobacterial multidrug treatment. There is a statistically significant difference on lung function and BMI between patients with NTM and NTM-PD. Improvements in lung function and nutritional status after treatment haven’t reached the statistical significance within the NTM-PD group. The average duration of therapy was 407 (68-609) days with an adverse effects incidence of 55%. Two patients interrupted treatment for serious side effects. In NTM-PD group 78% of patients underwent chronic therapy with azithromycin versus 35% of patients with NTM infection and 38% of NTM-PD patients had more than two pulmonary exacerbations the year before NTM isolation against 29%.

**Conclusions**

Prevalence of NTM-positive cultures in our sample is consistent with the literature. The radiological progression of the disease in NTM-PD despite anti-mycobacterial treatment and the considerable drug toxicity in CF adult patients highlights the need for a close monitoring of this population.

## P17 Importance of therapeutic compliance in Cystic Fibrosis (CF). Different evolution of chronic infection with MRSA: description of two case reports

### Mirella Collura^1^, Francesca Ficili^1^, Lisa Termini^1^, Maria A Orlando^1^, Gabriella Traverso^1^, Federica Arcoleo^**2**^, Tiziana Pensabene^**3**^, Marcella Bertolino^**1**^, Maria A Calamia^**1**^**,** Annalisa Ferlisi^**1**^

#### ^1^U.O. di Pediatria per la Fibrosi Cistica e le malattie respiratorie . ISMEP (Palermo), Palermo, Italy; ^2^Dipartimento di Scienze per la promozione della salute materno infantile G. D'Alessandro. Università degli studi di Palermo. Palermo, Italia; ^3^U.O. C. Microbiologia. ARNAS Civico Palermo. Palermo, Italia

##### **Correspondence:** Francesca Ficili (fficili@hotmail.com)

**Background**

Methicillin-resistant Staphylococcus aureus (MRSA) represents one of the most important new microrganisms that interest patients with cystic fibrosis (CF); it also represents an increasing clinical problem with limited, not always effective, therapeutic options. Because chronic infection with MRSA can be associated with accelerated decline in lung function, its eradication is attempted in most CF centers today. We present two case reports in which chronic infection with MRSA shows two different evolutions.

**Case reports**

DM, male 38 years old, with a complete form of CF, chronic infection with MRSA and Pseudomonas Aeruginosa, CFRD, chronic kidney failure, severe osteoporosis, showed since the first MRSA colonization a decline in lung function, with frequent need of hospitalizations, he also developed multi-drug resistance and many adverse reactions to drug. The patient also showed a decline in life quality through the compilation of CFQ-R questionnaire. The patient had always showed a successful adherence to antibiotics therapy and an intensive chest physiotherapy, with great limitations because of multiple adverse reactions to antibiotics with severe symptoms, so it was not always simple to treat lungs exacerbations and we treated him with antibiotics tested in sputum antibiogram. In the last year he was successfully admitted to the hospital for a lung transplant. FC,16 years old, with a complete form of CF, chronic infection with MRSA and also Pseudomonas Aeruginosa, showed since the first colonization from MRSA a decline in lung function, many drug resistance including also resistance to Linezolid, presents many lungs exacerbations, with a great need to hospitalization (10 hospitalization in the last 12 months ). It was not always simple to treat lung exacerbations and we treated him with antibiotics tested in sputum antibiogram. The second patient had not always an optimal adherence to all therapies concerning CF, first of all chest physiotherapy and use of inhaled antibiotics. The patient showed a deep decline of life quality through the compilation of CFQ-R questionnaire because of recurrent hospitalization and lungs exacerbations. Both the patients showed the same clinical and bacteriological situation instead of the presence in the first patient of multiple comorbidity such as CFRD, chronic kideys failure and osteoporosis.

**Conclusion**

We want to underline the importance of a correct adherence and compliance to all the therapies concerning CF. This behaviour lead to an improvement of clinical conditions, but also to an improvement of quality of life.

**Consent for publication:** The patients gave the consent to publish clinical data.

## P18 Time-resolved metagenomic identifies key features in the co-evolution of bacterial communities and Cystic Fibrosis

### Annamaria Bevivino^1^, Giovanni Bacci^2^, Federica Armanini^3^, Giovanni Taccetti^4^, Vincenzina Lucidi^5^, Daniela Dolce^4^, Patrizia Morelli^6^, Ersilia V. Fiscarelli^5^, Nicola Segata^3^, Alessio Mengoni^2^

#### ^1^ENEA Casaccia Research Center, Sustainable Territorial and Production Systems Department, Rome, Italy; ^2^University of Florence, Department of Biology, Florence, Italy; ^3^University of Trento, Centre for Integrative Biology, Trento, Italy; ^4^Cystic Fibrosis Center, Anna Meyer Children's University Hospital, Department of Pediatrics Medicine, Florence, Italy; ^5^Children's Hospital and Research Institute Bambino Gesù, CF Microbiology and CF Center, Rome, Italy; ^6^Cystic Fibrosis Center, G. Gaslini Institute, Department of Pediatrics, Genoa, Italy

##### **Correspondence:** Annamaria Bevivino (annamaria.bevivino@enea.it)

**Backgrounds**

Cystic fibrosis (CF) is characterized by chronic airway infections composed of polymicrobial communities. Though several studies have investigated the taxonomic composition of airways microbiota, little is still known about the genetic composition and function of CF airways microbial communities, and their relationship to disease benchmarks. In this study, we performed a time-resolved analysis of functional and taxonomic feature of lung microbiota in order to identify the key microbiome signatures associated with the increased risk of adverse outcomes and understand how microorganisms evolve in response to changes in clinical conditions along time.

**Materials and methods**

Twenty-one subjects with CF were enrolled in this study and followed over a 15-month period. Sputum samples were taken every three months during clinic visits. Patients were monitored during and after exacerbation. Library construction and metagenomic sequencing were performed following standard pipelines in Illumina Hiseq 2000 platform.

**Results**

An extraordinary resilience of the main CF pathogens to antibiotic treatment was detected. Hierarchical clustering based on microbial strain-level profiling of marker genes detected from metagenomics samples produced one cluster for each patient, containing all time points including those sampled through one or more exacerbation events. This effect was more evident for the main CF pathogens detected such as *Pseudomonas aeruginosa* and *Staphylococcus aureus*, but also for species than can be considered as emerging CF pathogens, such as *Rothia mucillaginosa* and *Prevotella melaninogenica*. Taxonomy distribution inferred from metagenomics data was quite heterogeneous both across patients and within time points of the same patient with some species that were not detectable especially during recovery, probably due to the antimicrobial treatment that might have drastically reduced the abundance of pathogen species below the revelation threshold.

**Conclusions**

Our results revealed that the airway colonization is highly selective and that a single strain, which started it, continues to survive and thrive in the lung of the patient. The possibility to analyze the microbiome dynamics in CF airways will permit to discover novel biomarkers involved in the pulmonary disease dynamics and can give us a set of tools to unlock the potential of microbiome-based personalized medicine in major disease areas including CF.

**Acknowledgements**

This research was financially supported by grants from “Fondazione Ricerca Fibrosi Cistica - ONLUS”: grant FFC#14/2015, with the contribution of “Delegazione FFC Fibrosi Cistica di Latina”, “Gruppo di Sostegno FFC Valle Scrivia Alessandria” and “Nonno Nanni Latteria Montello”, and grant FFC#19/2017.

## P19 Italian Cystic Fibrosis Registry (ICFR): Report 2011 – 2014

### Barbara Giordani^1*^, Annalisa Amato^1^, Fabio Majo^2^, Gianluca Ferrari^3^, Serena Quattrucci^4^, Laura Minicucci^5^, Rita Padoan^6^, Giovanna Floridia^7^, Gianna Puppo Fornaro^1^, Domenica Taruscio^3^, Marco Salvatore^3^

#### ^1^Lega Italiana Fibrosi Cistica ONLUS, Roma, Italia; ^2^Unità Operativa Complessa Fibrosi Cistica Ospedale Pediatrico Bambino Gesù, Roma, Italia; ^3^Centro Nazionale Malattie Rare, Istituto Superiore di Sanità, Roma, Italia; ^4^Centro di Riferimento per la Fibrosi Cistica – Regione Lazio, Roma, Italia; ^5^Centro di Riferimento per la Fibrosi Cistica – Regione Liguria, Genova, Italia; ^6^Centro di Supporto per la Fibrosi Cistica – Regione Lombardia, Brescia, Italia; ^7^Pre-BIO-Unità di bioetica, Istituto Superiore di Sanità, Roma, Italia

##### **Correspondence:** Barbara Giordani (gonexa@gmail.com)

**Background**

The Italian Cystic Fibrosis Registry (ICFR) is based on a new agreement (October 2016) signed by Istituto Superiore di Sanità (National Center for Rare Diseases), clinicians of the Italian National Referral and Support Centers for Cystic Fibrosis, Bambino Gesù Children's Hospital, Italian Cystic Fibrosis Society and Lega Italiana for Cystic Fibrosis.

**Materials and methods**

Analyses and results are referred to patients in charge to the Italian National Referral and Support Centers for Cystic Fibrosis in 2011-14 periods. Data were sent by Centers by means of specific software (Camilla, Ibis Informatica). Data underwent to a double quality control (QC): the first by Istituto Superiore di Sanità (ISS) and the second at a European level (European Cystic Fibrosis Registry). These QCs assure the completeness and accuracy of data as well as their consistency with European core data.

**Results**

A total of 29 different CF Centers sent their data to ICFR. Data regarding Sardinia are missing and those from Molise CF Center are exclusively referred to 2014. Estimated CF prevalence was 8,2/100,000 residents in Italy. On average, over the period 2011-2014, 52,1% of patients were male and 53,7% were aged more than 18 years. The majority of patients aged 7 to 35 years. Most of the CF patients were diagnosed before two years of age (around 66%); a significant percentage of patients (12%) was diagnosed in adult-age. In almost all patients, 2 (or more) CFTR mutations were identified. F508del mutation was the most frequent (44,8% in 2014). 135 patients received double lung transplantation (range 7-53 years; median age at transplantation was 32,5 years). Median waiting time for transplantation was 11 months. A total of 176 patients (median age 32 years; 81 males and 95 females) died in 2011-14 period.

**Conclusions**

Data from ICFR show that CF population is growing. A very low percentage of paediatric population is characterized by a severe impairment of FEV_1_; adult patients are characterized by an increase of age at death (more than 30 years).

## P20 Multidisciplinary approach in cf: a case report of two siblings

### Maria V Di Toppa^1*^, Nicoleta Popa^1^, Federico Alghisi^1^, Francesco Felicetti^1^, Sonia Graziano^2^

#### ^1^Cystic Fibrosis Unit, Bambino Gesù Pediatric Hospital, Rome, Italy; ^2^Clinical Psychology Unit, Bambino Gesù Pediatric Hospital, Rome, Italy

##### **Correspondence:** Maria V Di Toppa (mariavittoria.ditoppa@opbg.net)

**Background**

Cystic Fibrosis (CF) is a potential source of distress and there is growing recognition that the child’s care must focus on medical therapies and on the psychosocial well-being of patients and their families. We will describe a case report of two siblings that highlights the importance of integrate psychosocial care and standard medical practice. The case underlines the importance of a multidisciplinary approach to achieving global patient well-being.

**Materials and Methods**

Marco and Francesca come to our medical attention in 2014 for a "second opinion" after being taken care of at another service. They showed pancreatic insufficiency, Pseudomonas Aeruginosa colonization and frequent pulmonary exacerbations. Marco presented: FEV_1_= 70%; food allergy (milk, egg, gluten and nuts) and a severe malnutrition (weight 26 Kg, height 133 cm, BMI = 14.7) that required the placement of a Percutaneous Endoscopy Gastrostomy (PEG). Francesca presented: FEV_1_= 66%; (weight 36 Kg, height 143 cm, BMI = 17.6). They showed psychosocial problems (anxiety and depression symptoms, parental conflict after separation, poor knowledge of disease and poor adherence to treatment). The two brothers were followed up through a multidisciplinary approach that envisaged: a) medical care (hospitalization and day hospital); b) educational sessions about the use and maintenance of the devices, the management of therapy and proper nutrition; c) individual and family psychological support; d) multidisciplinary and systematic meetings of the medical team (doctors, nurses, psychologists, physiotherapists, social workers and dieticians) in order to identify common strategies.

**Results**

To date we have found: an improvement in Marco's nutritional state (weight 44.00 Kg, height 156 cm, BMI = 18.08%) moreover, the FEV1 values over these three years have reached 84%, thanks to the commitment of the boy in adhering to the care offered by the care team. Francesca also showed an improvement in lung occlusion with a FEV1 89%, increasing compared to its entry into the center; the nutritional state has improved (weight 49.2 Kg, height 155 cm, BMI = 20.4%). In both it has been detected an improvement compared to: nutritional status, better control of pulmonary infections, psychological functioning, awareness of the disease, adherence to therapeutic treatment emotional and social adaptation and coping skills, improving global health and quality of life. Both have joined the center's telemedicine project by regularly broadcasting their values of saturation and spirometry.

**Conclusions**

A multidisciplinary approach in a complex chronic condition, such as CF, appears to be a necessary condition for achieving goals that promote the overall well-being of the patient.

**Consent for publication**

*I declare that I have obtained the consent to publish the clinical data of the two patients. I declare that I have used fantasy names.*

## P21 Patient engagement in Cystic Fibrosis during the therapeutic process

### Riccardo Ciprandi^1^, Rita Pescini^2^, Guendalina Graffigna^1^, Serena Barello^1^, Rosaria Casciaro^2^, Federico Cresta^2^, Laura Minicucci^2^

#### ^1^Università Cattolica del Sacro Cuore, Milano, Italia; ^2^Istituto Giannina Gaslini, Genova, Italia

##### **Correspondence:** Rita Pescini (ritapescini@gaslini.org)

**Background**

Patient Engagement should be considered as a key priority to innovate healthcare services delivery in the context of Cystic Fibrosis (CF), a chronic severe disease. The objectives of this study have been to identify pivotal variables associated to a greater Patient Engagement in patients with CF and to investigate the management of the disease.

**Materials and methods**

This is a cross-sectional study involving a sample of 60 patients affected by CF. The survey featured 4 validated scales. Particularly the following measures were used to indagate the level of: Patient Engagement (PHE-S), Patient Activation (PAM), Self Efficacy (SE), Positive and Negative Affect States (PANAS). Data were analysed performing Bivariate Correlations.

**Results**

We have conducted the survey in a sample composed by 29 males and 31 females, mean age 26,5 (±10,55); mean BMI 20,76 (±2,67); also 37,7% (n=23) featured exacerbations in the last year and 53,3% (n=32) takes from 5 to 15 drugs. We have measured the level of Patient Engagement in this sample and 20% (n=12) is in the Arousal phase: they are starting to act; 60% (n=36) in the Adhesion phase: they have learned behavioural skills to act in a proactive manner towards the disease. The last 20% (n=12) is in the Eudaimonic project phase: they have fully accepted the disease and they are good stakeholder for a positive engagement. We have measured also the Patient Activation and 50% of the sample (n=30) results to be in a phase of full activation: the patients have realised most of the needed behavioural changes. The last interesting outcome observed is a low adherence to pharmacological therapies in 55% (n=33) of the sample. Furthermore we have analysed the correlations between Patient Engagement and the aforementioned variables (Table 8).

**Conclusions**

The study highlights the correlations among Patient Engagement and some important variables: Patient Activation, Self Efficacy and Negative Affect States. These factors are essential to promote a positive engagement. In closing, we have to notice that the low adherence to therapies suggests to improve an effective communication between patient and clinician.

**Table 8:** **(abstract P21).** Patient Engagement correlations

Correlations	R	p
Patient Engagement - Patient Activation	0,593	0,000
Patient Engagement -Self Efficacy	0,538	0,000
Patient Engagement -Negative Affect States	-0,569	0,000

It is important to underline the inverse correlation between Patient Engagement and Negative Affect States: a good engagement implies a low level of negative affect states

## P22 How and to what extent we can know our patient’s emotional state?

### Paola Catastini^1^, Salvatore De Masi^2^, C. Braggion^1^

#### ^1^CF Centre Meyer Children’s Hospital, Florence, Italy; ^2^Clinical Trial Office Meyer Children’s Hospital, Florence, Italy

##### **Correspondence:** Paola Catastini (catastini@iol.it)

**Background**

In cystic fibrosis (CF) the daily treatment is inevitable and affects the patient’s emotional state.

TIDES study showed a higher rate of anxiety and depression, underlying how these symptoms are common in CF. Untreated anxiety and depression can have an impact on adherence and patient’s quality of life. Our study evaluates prevalence of depression and anxiety with new questionnaires, according to International Guidelines, and the relationship of emotional states with the quality of life.

**Materials and Methods**

113/183 (61.7%) adult patients (64 males, mean age 34.8 ± 9.7 years) followed at our Centre were screened by the PHQ-9 (Patient Health Questionnaire-9) and GAD-7 (Generalized Anxiety Disorders - 7 item) to investigate depression and anxiety scores, respectively. The CFQ-R questionnaire was also submitted. Patients agreed to personal data treatment.

**Results**

The PHQ-9 score was in the normal range (0-4) in 62 patients (54.9%), between 5 and 9 (mild depression) in 33 patients (29.2%), and > 10, suggesting a moderate-severe depression in 18 patients (15.9%). Considering the GAD-7 questionnaire, we found a normal score in 63 patients (55.8%), a score suggesting a mild anxiety in 33 patients (29.2%) and a score suggesting a moderate-severe anxiety in 17 patients (15.0%). We found a negative correlation between anxiety score and all domains of CFQ-R, except for physical and respiratory domains and between depression score and all domains of CFQ-R, except for respiratory domain.

**Conclusions**

According to the CFF and ECFS consensus statements about a third of our population should receive a supportive intervention and psychoeducation, and about 15% of our adults should have a psychological and/or psychopharmacological intervention. When we used the HADS scale in a different group of CF subjects, we found a similar prevalence of elevated, > 10, anxiety (17.2 vs 15.4%) but a lower prevalence of elevated, >10, depression (6.6 vs 15.9%) in comparison with the data obtained in this study. We wonder if the proposed modality of screening and treatment for anxiety and depression is also applicable in Italy. Two aspects are critical: i) there is a very small number of psychologists, who works full-time in the Italian CF Centers; ii) the psychological intervention is more directed to improve the coping of patient-family with the disease during specific life times (diagnosis, growth and nutrition, adolescence, self-care and independence, worsening of disease and decisions on organ transplantation, end of life issues).

## P23 Cystic fibrosis is a known disease?

### Maria A Calamia^1^, Annalisa Ferlisi^1^, Maria G Silvestro^1^, Lucia Guarnuto^1^, Francesca Ficili^1^, Emanuela Di Liberti^2^, Valentina Patti^1^, Mirella Collura^1^

#### ^1^U.O. II Pediatria per la Fibrosi Cistica e le malattie respiratorie. ISMEP – Palermo, Italia; ^2^Università degli studi di Palermo, Palermo, Italia

##### **Correspondence:** Francesca Ficili (fficili@hotmail.com)

**Background**

We present an observational study made to know how cystic fibrosis (CF) is known by two samples: nurses population and society.

**Materials and Methods**

Every sample was investigated using a questionnaire. It consists of 18 multiple choise questions, submitted to two samples: the "Nurses" sample, consisting of 50 nurses belonging to different wards not concerning CF and the "Society" sample, including 50 subjects who don't study or work in the healthcare field. Questions was aimed to investigate the CF knowledges about incidence, symptoms, diagnosis, treatment and life expectancy and to understand if the samples know the existence of the CF Center and which healthcare professionals are involved in the patients care.

**Results**

The results suggest that: 18% of nurses surveyed and 68% of subjects belonging to the "Society" sample don't know CF; 72% of nurses interviewed and 90% of subjects belonging to the "Society" sample don't know people with CF; 2% of nurses and 48% of the "Society" sample say that CF is not a genetic disorder; 70% of subjects in both samples think that CF is a very rare disease; 28% of subjects belonging to the “Society” claim that patients with CF are contagious; 22% of surveyed nurses and 35% of the "Society" sample don't know the organs involved, symptoms, diagnosis and treatments; very often CF is being exchanged for a neurodegenerative/neuromuscular disease; 64% of the two sample don't recognize that life expectancy is greatly improved by the advancement of scientific research, the implementation of new treatments, and the improvement of care processes. Almost 58% of the "Society" sample dont' recognize the existence of the CF Center, which is dedicated to the care and assistance .14% of nurses interviewed and 22% of subjects belonging to the "society" sample doesn't know that there is a CF Center also in Palermo; 14% of nurses surveyed and 36% of subjects belonging to the "Society" sample are not interested in learning more about CF.

**Conclusions**

The results obtained show that this disease it's not so well-known among two sample due to poor media involvement and also probably due to poor university and post-based training on. These results must make us reflect on the role that healthcare professionals have in educating society about the knowledge of this pathology. It's necessary to increase the awareness of the scientific world and not to give the right attention to CF.

## P24 Analysis of disease experience in patients with Cystic Fibrosis to support adherence to long-term therapies

### Piercarlo Poli, Valentina Tradati, Rita Padoan

#### Cystic Fibrosis Support Center, Department of Pediatrics, Children’s Hospital, ASST Spedali Civili, Brescia, Italy

##### **Correspondence:** Piercarlo Poli (piercarlo.poli@gmail.com)

**Background**

Rigorous systematic reviews have found that in developed countries, adherence to long-term therapies among patients with chronic disease is only 50% [1].

Cystic fibrosis (CF) is a genetic disease that affects several organs, including the respiratory, digestive and reproductive systems, but above all is a "time-consuming" disease.

CF requires complex medical management throughout the patient's life. The therapeutic load becomes increasingly intense with the course of the years and with the worsening of the disease and it is known that adherence to therapies dramatically decreases since adolescence, if not before.

Adherence between children and adults with CF varies from 38% for chest physiotherapy to 50% for inhalation therapies and dietary supplements [2] and 68% for the new drug Kalydeco©[3]. Talking about adherence to therapy during an outpatient visit is not always easy and it is not always done. Riekert et al provided a questionnaire to US CF Foundation accredited CF centers, showing that only 8% of respondents measure adherence, and only 64% discuss about adherence during the outpatient follow-up [4]. The most common used strategy to increase adherence of CF patients is disease education.

**Materials and methods**

We analyzed the disease awareness of 79 patients older than 8 years followed at the Regional Support Center for CF in Brescia. For each age group (8-11, 12-17,>18) an electronic questionnaire (Google Forms©) was created. Questionnaires were subdivided into thematic sections, each provided with several multiple choice or short answer questions. The topics covered are as follow: knowledge of the disease and self-management, knowledge about health, infection control, chest physiotherapy, lifestyle, psychosocial well-being, sexual education, professional/educational plan and social care.

**Results**

Results are reported in Table 9.

**Conclusions**

Below are the key points of educational intervention based on the outcome of our patients' needs:

- Patient education starts at the time of diagnosis and must be integrated into each stage of the evolution of the disease.
- All members of the multidisciplinary team must contribute to patient education.
- Instructing the patient to self-management of the disease, adapting the information and therapy according to his/her needs.
- Developing therapeutic plans in collaboration with all members of the team and patients.
- Encourage adherence to the therapeutic regimen.
- Monitor adherence.

**References**

1. Haynes RB, McDonald H, Gang AX, et al. Interventions for helping patients to follow prescriptions for medications. Cochrane Database Syst Rev, 2002(2): CD000011.
2. Arias Llorente RP, Bousono Garcia C, Diaz Martin JJ. Treatment compliance in children and adults with cystic fibrosis. J Cyst Fibros. 2008;7:359-67.
3. Siracusa CM, Ryan J, Burns L, et al. Electronic monitoring reveals highly variable adherence patterns in patients prescribed ivacaftor. J Cyst Fibros. 2015;14:621-6.
4. Riekert KA, Eakin MN, Bilderback A, et al. Opportunities for cystic fibrosis care teams to support treatment adherence. J Cyst Fibros. 2015;14:142-8.

**Table 9:** **(abstract P24).** Examples of the answers from the questionnaire analysis

Age group	8-11		12-17		>18	
	Yes	No	Yes	No	Yes	No
% of patients responding to the questionnaire	63%	37%	91%	9%	89%	11%
Seasonal flu vaccination	100%	0%	61%	39%	67%	22%
Basic knowledge about CF related diabetes	//		32%	68%	68%	32%
Basic knowledge about lung transplantation	//		//		41%	59%
Do you sleep properly?	//		93%	7%	67%	33%

## P25 DRESS syndrome in a child with Cystic Fibrosis

### Massimo Luca Castellazzi, Valeria Daccò, Laura Claut, Carla Colombo

#### Cystic Fibrosis Centre, Fondazione IRCCS Ca' Granda, Ospedale Maggiore Policlinico, University of Milan, Milan, Italy

##### **Correspondence:** Massimo Luca Castellazzi (lucastellazzi85@gmail.com)

**Background**

Drug reaction with eosinophilia and systemic symptom (DRESS) syndrome is a rare life-threatening hypersensitivity reaction with an estimated incidence between 1:1000 and 1:10000 drug exposures occurring 2-6 weeks after a causative therapy. DRESS syndrome is characterized by skin eruption, fever, elevated liver enzymes and leukocytosis with eosinophilia. Renal, pulmonary, cardiologic and neurologic manifestations may be also associated. This condition may be fatal if unrecognised, especially in patients with hepatic failure. Diagnosis may be difficult, particularly in paediatric and cystic fibrosis (CF) patients in which this condition is rarely described.

**Case report**

A 4-year-old girl with CF admitted to our hospital for a pulmonary exacerbation was treated with piperacillin-tazobactam (150 mg/kg/die) and tobramycin (10 mg/kg). After 14 days she developed fever (up to 40°C) and a diffuse maculopapular erythematous rash. Generalized polyadenomegaly and hepatomegaly were also detected. Laboratory investigations revealed a marked increase in C-reactive protein, lactate dehydrogenase, aspartate and alanine aminotransferse and a significant alteration in coagulation tests. On the 18^th^ of hospitalization hypereosinophilia was also observed (Table 10). Autoimmune, infective and hematologic detection tests were negative.

Due to the clinical features the diagnosis of DRESS syndrome was performed and the ongoing antibiotic treatment discontinued. Without others therapies, clinical resolution was progressively achieved.

**Conclusions**

CF patients have an increased incidence of adverse drug reactions due to the heavy therapeutic burden they are exposed in order to prevent progressive lung damage. To our knowledge, this is the first report of DRESS in a paediatric patient with CF. Due to its highly variable clinical presentation, DRESS may be difficult to diagnose as it can mimic infections, autoimmune diseases, hematologic and lymphocytic disorders. Our case highlights the importance to be aware of this condition as the removal of the offending drug is the main management step for the resolution of the clinical and laboratory features.

**Consent for publication:** The authors confirm written informed consent was obtained from the patient’s parents.

**Table 10:** **(abstract P25).** Characteristics of patients

Laboratory data	DAY 1	DAY 14	DAY 18	DAY 28
WBC (4.800-12.100/mmc)	5.600	8.320	12.140	7.820
Eosinophils (100-500/mmc)	220	30	2940	440
CRP (<0.5 mg/dl)	0.59	10,31	3,19	0,14
AST-ALT (5-36 U/l e 5-29 U/l)	31-28	402-62	1560-311	36-60
LDH (120-300 U/l)	276	3637	10880	300
PT-aPTT (0.94-1.22 e 0.86-1.20)	-	-	1,23-1,94	0,96-1,03
D-dimero (<230 ng/ml)	-	-	68384	230

## P26 The support of the thoracic ultrasound in the reexpansion techniques of a lung parenchymal atelectasic area: case report

### Matteo Giuliari^1^, Luana Vicentini^1^, Fausto Tilotta^2^, Antonella Paciaroni^2^, Sabino Della Sala^2^, Cristina Guerzoni^1^, Elisa Andreatta^1^, Grazia Dinnella^1^

#### ^1^U.O. di Pediatria, Centro di Supporto provinciale per la cura della Fibrosi Cistica, Ospedale di Rovereto – Trento, Italy; ^2^U.O. di Radiologia, Ospedale di Rovereto – Trento , Italy

##### **Correspondence:** Matteo Giuliari (matteo.giuliari@apss.tn.it)

**Background**

Atelectasia is a non ventilated lung parenchyma area which is a possible complication of many lung diseases, including Cystic Fibrosis (CF). The treatment of this pathological event includes the use of reexpansion techniques through positive pressure devices, such as continuous positive airway pressure (CPAP) and facilitating postures. Atelectasia is detectable through diagnostic imaging examinations such as radiographic examination (RX), computed tomography (CT) and magnetic resonance imaging (MRI), which provide a static image of the involved parenchymal damage. An additional tool in the diagnostic imaging examinations is thoracic ultrasound (TUS), which in addition to the static image, is able to return a dynamic evaluation of the functional response of non-ventilated areas during breathing. This allows to evaluate in real time the ability of parenchyma to reexpand during the application of physiotherapy techniques.We describe a clinical case of pulmonary atelectasis in the lingular site, detected at MRI and treated with non invasive ventilation (NIV) for drainage and CPAP for reexpansion in posture during TUS monitoring.

**Case report**

Twenty four-year-old female patient with CF, admitted to Trentino Provincial CF Support Center for intravenous antibiotic therapy. On 10th day, MRI showed alteration of lung echoes with longitudinal and transverse extension of about 3 cm, compatible with atelectasic area at the lingular site. TUS was used at T0 to identify the atelectasia in order to set the application of physiotherapy techniques and at T1 (24 hours), as outcome measure. Physiotherapy program for bronchial drainage consisted of NIV (IPAP 13 cmH2O, EPAP 5 cmH2O), 3 times in 24 hours, followed by CPAP application for reexpansion, set at 8 cmH2O in semi-supine and right side facilitating posture, for 1 hour.

The patient allowed the personal data treatment. After the application of 3 physiotherapy sessions of bronchial drainage, followed by CPAP techniques, the small area compatible with atelectasia was almost no longer recognizable at TUS monitoring.

**Conclusion**

TUS is a complement to current imaging techniques that can help the respiratory physiotherapist to set, quantify and modify its treatment in real time. The exam is quick, non-invasive, with no side effects and can be performed even for bedridden patients. It requires trained medical personnel and a multidisciplinary team work.

**Consent for publication:** The patients gave the consent to publish clinical data.

## P27 Detection of false negative cases by cystic fibrosis screening: our experience

### Lisa Termini^2^, Valeria Pavone^1^, Elisa Parisi^1^, Francesca Ficili^2*^, Maria A Orlando^2^, Annalisa Ferlisi^2^, Gabriella Traverso^2^, Orazia M Granata^3^, Tommaso S Aronica^3^, Mimì Crapisi^3^, Donatella Fogazza^2^, Marcella Bertolino^2^, Mirella Collura^2^

#### ^1^Scuola di specializzazione in Pediatria. Università degli studi di Palermo, Palermo, Italy; ^2^U.O. di Pediatria per la Fibrosi Cistica e le malattie respiratorie. ISMEP, Palermo, Italy; ^3^U.O. Patologia clinica pediatrica. Screening neonatale metabolico allargato. ISMEP, Palermo, Italy

##### **Correspondence:** Francesca Ficili (fficili@hotmail.com)

**Background**

Cystic fibrosis (CF) is the most common recessive genetic disorder in Caucasian population. The suspicion of disease comes from the detection of typical symptoms, family history and newborn screening (NBS). NBS is the only tool that allows reaching an early diagnosis lowering disease severity, burden of care and costs. The first step is based on the dosage of Immunoreactive trypsin (IRT) on spot of blood taken between 3rd-5th day of life. Intermediate tiers are required to achieve an acceptable combination of sensitivity and specificity: second IRT test, CFTR mutation analysis. Sweat test is the gold standard used to confirm the diagnosis.

**Materials and methods**

The NBS protocol used in Palermo is IRT-IRT-DNA. If the first IRT is > 54 ng/ml a second test is made with the same sample to increase test accuracy. If is still > 58 ng/ml a second sample is collect at about 3-4 weeks and if results still high infants are involved for the genetic test. Detection of CFTR gene mutations defines the diagnosis and help to predict the phenotype. Positive NBS are followed by confirmatory sweat-test.

**Results**

Over a 12 months period (01/09/16-30/09/17) in Palermo we had 17 new cases of CF. Six cases were not screened because born before the introduction of NBS. Genetic test was performed in adulthood (two patient’s parents and four people with infertility). One case results unknown (performed abroad). Eleven cases were screened with four positive test. In six cases, the screening programme failed. One of our false negative case had meconium ileus. Another one had low haematocrit value that altered the result. The others were not detected due the high cut off value adopted. All these cases were suspected for symptoms potentially related to CF (hypocloremic metabolic alkalosis, dehydration, acute pancreatitis, meconium ileus, poor growth) but a sweat test and DNA study were delayed because of false reassurance from the fact that the child were screened.

**Conclusions**

False negativity can be associated with laboratory errors, bad choice of cut-off values, pancreatic sufficiency, meconium ileus. The choice of the centile-cut-off affects the screening’s efficacy and is a compromise between the need for good accuracy and economic and social costs. Considering the high prevalence of false negativity, our centre is working to increase test sensitivity lowering the first IRT cut-off, to make a second test on the same sample (from 54 to 50 ng/ml) and defining the second IRT cut-off 40 ng/ml at 3-4 weeks of age.

## P28 A premature infant with Cystic Fibrosis: case report

### Mirella Collura^1^, Luca Alessi^2^, Flavia Mulè^2^***,*** Marcello Vitaliti^3^, Mariarosaria Maresi^3^, Maria A Orlando^1^, Annalisa Ferlisi^1^, Gabriella Traverso^1^, Lisa Termini^1^, Marcella Bertolino^1^, Francesca Ficili^1^

#### ^1^U.O. di Pediatria per la Fibrosi Cistica e le malattie respiratorie. ISMEP, Palermo, Italy; ^2^Scuola di specializzazione in Pediatria. Università degli studi di Palermo, Palermo, Italy; ^3^Neonatologia con UTIN e Nido. ARNAS Civico. Palermo, Italy

##### **Correspondence:** Francesca Ficili (fficili@hotmail.com)

**Background**

To report the clinical course of a preterm infant with diagnosis of cystic fibrosis.

**Case report**

A female infant was born via cesarean section for podalic presentation secondary to premature onset of labor, weighing 1140 g at 28 weeks and 3 days of gestational age, with Apgar scores of 3 and 7. Her mother had attended many prenatal consultations with negative serology for vertical infections and normal obstetrical ultrasound. Immediately after birth, the infant was referred to the neonatal critical care unit due to early respiratory distress, abdominal distension and frequent regurgitations. Serial abdominal radiographs revealed air fluid levels. Initially she was stabilized with nasogastric and rectal decompression. In the tenth day of life the patient underwent laparotomy surgery for intestinal occlusion/ meconium ileus. Ileal resection and two ileostomies were performed, followed by reconstruction of the bowel transit at 17 days of life. She had late neonatal sepsis during the hospital stay, requiring prolonged antibiotic therapy. She was intermittently ventilator dependent, secondary to multiple surgical procedures and sepsis. Two transfusions of red blood cells were performed. Parenteral nutrition was gradually transitioned to infant formula and pancreatic enzyme supplements with satisfactory weight gain. On the basis of her complicated clinical course, Cystic Fibrosis Transmembrane Conductance Regulator (CFTR) mutation analysis had been performed using the Cystic Fibrosis Genotyping Assay (Celera-Abbott). Mutation analysis showed homozigosity for the common CFTR mutation [delta]F508. The sweat chloride test was carried out with a positive result (89 mEq/L). At three months her chest X-ray revealed left pulmonary hyperinflation and right pulmonary consolidation compatible with pneumonic disease. After culture, and based on the microorganism isolated from the upper respiratory tract secretion (Klebsiella pneumoniae and Escherichia coli), antibiotic therapy was prescribed. After prolonged hospitalization, she was discharged home, but after few days she was hospitalized again for bronchiolitis. Laboratory tests for respiratory syncytial virus was positive. During hospitalization, due to progressive worsening of the respiratory pattern, noninvasive ventilation (HFNC) was applied for seven days. She was discharged home on room air and she has not been hospitalized anymore. Ongoing she receives continuous care from paediatric cystic fibrosis centre, has a good nutritional status taking infant formula with pancreatic enzymes supplementation and performs PEP-mask physiotherapy with benefit.

**Conclusion**

This case report highlights the importance of advanced neonatal care with cystic fibrosis therapy, and in particular the joint management of two care units and of appropriate treatment of bronchiolitis in CF patient.

**Consent for publication:** Patient’s parents signed consent for the publication of patient’s clinical data.

## P29 Efficacy and safety of Kalydeco oral granules: the experience of our centre

### Fabiola De Gregorio, Antonella Tosco, Alida Casale, Angela Sepe, Chiara Cimbalo, Andrea Catzola, Alice Castaldo, Laura Salvadori, Valeria Raia

#### Cystic Fibrosis Center, Department of Translational Medical Sciences University of Naples “Federico II”, Naples, Italy

##### **Correspondence:** Fabiola De Gregorio (fabioladegregorio@yahoo.it)

**Background**

Ivacaftor (Kalydeco, Vertex Pharmaceuticals) is the first CFTR potentiator approved for the treatment of cystic fibrosis (CF) patients carrying at least one gating mutation. On January 2016 AIFA approved KALYDECO oral granules for patients aged 2-5 years/weight <25Kg. We report our experience with patients in treatment with Kalydeco oral granules.

**Materials and methods**

We evaluated patients treated with Kalydeco oral granules since 2016. Patients underwent visit every 4 weeks for the first 3 months, then every 3 months. Sweat test was performed within 14 days of therapy. The following data were registered each visit: sweat Cl, weight, height, BMI, vital signs, physical examination, renal, hepatic and pancreatic profile, spirometry and six-minutes walking test (6MWT) (if available), sputum/pharyngeal aspirate colture. Ophthalmological and TC examination (sinus and lung) was performed at baseline and after 6 months and 1 year of treatment, respectively. CFQR was administered at baseline, after 8 weeks and 24 weeks of therapy, then every 24 weeks. Each adverse event was noted.

**Results**

Four patients, mean age 5.6 years (range 3.6-8.3), 3 F, started therapy with Kalydeco oral granules. CFTR gene analysis showed the following mutations: 2/4 G1244E, 1/4 S549N, 1/4 G178R. 2/4 had pancreatic sufficiency. 2/4 had history of recurrent acute pancreatitis with high amylase (AMS) and lipase (LPS) values at baseline in 1/2. Mean age at baseline was 4.9 years (range 3.1-7.9). Mean follow-up was 34 weeks (range 9-62). Mean Cl value at baseline was 78.5 mEq/l (range 67-86). Through the treatment Cl values decreased <60mEq/l since day 14. Weight increases in 3/4 patients. No pulmonary exacerbations were observed, except in 1 patient. Only in 1 patient change in FEV1, 6MWT and CFQR was evaluated with an increase in FEV1 of 9.67% and in CFQR respiratory score of 8.3 at week 8. Only 1 patient performed TC exam: no bronchiectasis and no change from baseline were reported. None of patient registered change in serum chemical and hematologic tests, in faecal elastasis and sputum culture. Normalization of AMS e LPS was observed in the patient with pancreatitis. Headache, upper respiratory tract infection, nasal congestion occurred in 2/4. No ocular anomalies were detected.

**Conclusions**

Ivacaftor has been confirmed as an effective and safe drug, even in our small cohort of young patients. Further long term observation is needed to evaluate a significant clinical improvement of other important disease features as glucose and bone metabolism.

**Consent for publication:** Written informed consent was obtained from the patient’s parents.

## P30 Effects of Ivacaftor in two patients with Cystic Fibrosis and severe lung disease carrying CFTR splicing mutations

### Donatello Salvatore, Carmela Colangelo, Giovanni Marsicovetere, Michele D’Andria, Domenica Passarella, Carmela Genovese

#### Centro Regionale Fibrosi Cistica, AOR Ospedale San Carlo, Potenza, Italy

##### **Correspondence:** Donatello Salvatore (saverdon@gmail.com)

**Background**

Ivacaftor is a CFTR potentiator that corrects chloride transport in most CFTR class III mutations affecting channel gating, some class IV mutations exhibiting abnormal gating or conductance defect and several missense mutations associated with defects in protein processing or function. Ivacaftor is globally approved for class-III and *R117H* CFTR mutations and, in the United States, for further 28 mutations (23 missense mutations and 5 splicing-affecting mutations) resulting in partially functioning CFTR protein. The *3272-26A*→*G* variant is a class V mutation. Phenotype associated to this variant is variable, with either mild and severe lung disease described; pancreatic sufficiency (PS) is frequent. The *3849+10kbC*→*T* variant is also a class V mutation. Phenotype of patients having the *3849+10kbC*→*T* variant is highly variable, with lung disease delayed in onset in most of these patients, but then become severe in some. The majority of patients have PS, borderline or normal sweat chloride (SCL) values and sometimes male fertility. We describe the effectiveness and safety of Ivacaftor in two women with CF with the CFTR genotype *3272-26A*→*G /E585X and 3849+10kbC*→*T /F508del*, respectively, with severe lung disease.

**Materials and methods**

Ivacaftor 150 mg bid was started after a request of compassionate use was approved by the local Ethics Committee (EC). Before starting the therapy with Ivacaftor, a basal assessment was performed (lung function, 6-minute walking test (6MWT), CFQ-R questionnaire, sweat test, biochemical tests, microbiology test, ophthalmologic evaluation). Data about lung function, 6MWT, antibiotic therapies were retrieved by the local database. Subjects were admitted as outpatients once a month.

**Results**

Main results are summarized in Table 11. The administration of Ivacaftor resulted in improvement of lung function, 6MWT, CFQ-R scores and a decrease of antibiotic therapy in both patients. Sweat chloride decreased only in the patient with 3849+10kbC→T/F508del genotype, whereas nutrition and sputum microbiology were unchanged. No safety concerns were registered for both patients.

**Conclusions**

These cases expand our knowledge about potential benefits of Ivacaftor for CFTR mutations with RF, including certain mutations affecting splicing. The clinical and functional improvement in subjects with severe lung damage suggests that Ivacaftor could alter the natural history of the disease, when an adequate target is present, as a CFTR with altered gating function or defective conductance or a quantitative partial defect of CFTR, as in presence of mutations affecting splicing.

**Consent for publication:** The patients gave the consent to publish clinical data.

**Table 11:** **(abstract P30).** Characteristics of the patients and main results

	Patient 1	Patient 2
Current age (years)	32	47
Gender	Female	Female
Genotype	*3272-26A*→*G /E585X*	*3849+10kbC*→*T /F508del*
Sweat chloride (mmol/L) Before Ivacaftor	82	57
After Ivacaftor	83 (6 and 18 months)	19 (6 months)
FEV_1_ (% PREDICTED) Before Ivacaftor	29	38
After Ivacaftor	44 (6 months); 52 (18 months)	50 (6 months)
6MWT (meters) Before Ivacaftor	500	508
After Ivacaftor	630 (6 and 18 months)	572 (6 months)
Days of antibiotic therapy 12 months Before Ivacaftor	152 (67 IV and 85 oral)	88 (36 IV and 52 oral)
After Ivacaftor	49 (18 months) oral	0 (6 months)
CFQ-R Respiratory Domain	33 (Basal) 78 (18 months)	72 (Basal) 83 (6 months)

## P31 Patients in critical need in Ivacaftor-Lumacaftor treatment: experience of Cystic Fibrosis Center of Palermo

### Mirella Collura^1^, Elisa Parisi^2^, Annalisa Ferlisi^1^, Mari A Orlando^1^, Gabriella Traverso^1^, Lisa Termini^1^, Marcella Bertolino^1^, Caterina Di Girgenti^3^, Maria A Calamia^1^, Maria G Silvestro^1^, Stefania Barrale^1^, Maria R Bonaccorso^1^, Annalisa D'Arpa^2^, Francesca Ficili^1^

#### ^1^U.O. Pediatria II per la FIBROSI CISTICA (CRR) e le Malattie Respiratorie – Allergologia– ISMEP, Palermo, Italy; ^2^Dipartimento di Scienze per la promozione della salute materno infantile G. D’Alessandro. Università degli studi di Palermo, Palermo, Italy; ^3^U.O.S.D. Genetica molecolare. ARNAS Civico, Palermo, Italy

##### **Correspondence:** Francesca Ficili (fficili@hotmail.com)

**Background**

Cystic fibrosis (CF) is a life-limiting disease that is caused by defective or deficient CF transmembrane conductance regulator (CFTR) protein activity. Phe508del is the most common CFTR mutation with high rates of premature death. It is a multisystem disease that is characterized by pancreatic insufficiency and chronic airway infections associated with loss of lung function, repeated pulmonary exacerbations. Lumacaftor is a CFTR corrector that has been shown in vitro to correct Phe508del CFTR misprocessing and increase the amount of cell surface–localized protein. Ivacaftor is an approved CFTR potentiator that increases the open probability of CFTR channels.

The TRAFFIC and TRANSPORT trials were two phase 3, multinational, randomized, double-blind, placebo-controlled, parallel-group studies in which Lumacaftor was orally administered combined with Ivacaftor.

**Materials and methods**

Between March 2016 and September 2017 at the Regional Reference Center of Palermo for the CF, a total of 7 patients in critical need (female:5, male:2) aged 17–44 years (mean 26,6) with CF, who were homozygous for the Phe508del, were enrolled and assigned to receive Lumacaftor/Ivacaftor. At first we evaluated auxometry (weight, hight and BMI), respiratory function (%FEV_1_ and %FVC), ECG, liver, pancreatic and renal function, sweat test, respiratory infections and CFQR. Patients agreed to personal data treatment.

**Results**

Significant improvements in the percentage of predicted FEV1 were seen, the respiratory function got better in term of FEV1 an FVC in this 18 month of observation: FEV1 + 11,4 % after 3 months (T3), + 12,2% after 6 (T_6_), +9,8 one year later (T_12_) and + 6,8 at the end of the observation (T_18_). Also the FVC increased of 10,4 %, 13%, 9,3% and 9,5% respectively after 3, 6, 12 and 18 months (T_3_, T_6_, T_12_, T_18_). We observed the improvement of the mean-BMI: +0,16 percentage points after six months (T_6_) and 0,21% at the end (T_18_) (Table 12). We recorded nine cases of pulmonary exacerbations and any side effects at the end, except for a patient who suffered of a subopacity of the crystalline. The sweat test results were not reliables. The quality of life was investigated by the Cystic Fibrosis Questionnaire-Revised (CFQ-R) respiratory symptom scale, that revealed an improved tolerance to effort and a decreased night bronchorrea.

**Conclusions**

The discovery of this therapeutic strategies gave new prospective of life to the patients who carried a Phe508del mutation in CFTR. Our data demonstrated, according to the international literature, that combination therapy of Lumacaftor/Ivacaftor improves the respiratory function and the quality of life of these patients.

**Table 12:** **(abstract P31).** Evaluation of FEV1, FVC, CFQR, BMI

	T_0_	T_3_	T_6_	T_9_	T_12_	T_15_	T_18_
FEV1 (%)	35,2	46,6	47,4	45	45	44,4	42
FVC (%)	51,4	61,8	64,4	63	60,7	63,4	63,6
CFQ-R (%) (Respiratory)	62	-	-	-	-	-	72,6
Mean-BMI (Kg/m^2^)	19,67	18,51	19,83	19,95	19,98	19,85	19,88

